# High Antiproliferative Activity of Hydroxythiopyridones over Hydroxypyridones and Their Organoruthenium Complexes

**DOI:** 10.3390/biomedicines9020123

**Published:** 2021-01-27

**Authors:** Md. Salman Shakil, Shahida Parveen, Zohaib Rana, Fearghal Walsh, Sanam Movassaghi, Tilo Söhnel, Mayur Azam, Muhammad Ashraf Shaheen, Stephen M. F. Jamieson, Muhammad Hanif, Rhonda J. Rosengren, Christian G. Hartinger

**Affiliations:** 1 Department of Pharmacology and Toxicology, University of Otago, PO Box 56, Dunedin 9016, New Zealand; salman.shakil@postgrad.otago.ac.nz (M.S.S.); ranzo073@student.otago.ac.nz (Z.R.); azaad833@student.otago.ac.nz (M.A.); 2 School of Chemical Sciences, University of Auckland, Private Bag 92019, Auckland 1142, New Zealand; shahidap2009@gmail.com (S.P.); fwal135@aucklanduni.ac.nz (F.W.); smov524@aucklanduni.ac.nz (S.M.); t.soehnel@auckland.ac.nz (T.S.); 3 Department of Chemistry, University of Sargodha, Sargodha 40100, Pakistan; mashaheen1964@gmail.com; 4 Auckland Cancer Society Research Centre, University of Auckland, Private Bag 92019, Auckland 1142, New Zealand; s.jamieson@auckland.ac.nz

**Keywords:** 3-hydroxy-4-pyridone, 3-hydroxy-4-thiopyridone, anticancer agents, apoptosis, cytotoxicity, Ru(arene) complexes

## Abstract

Hydroxypyr(id)ones are a pharmaceutically important class of compounds that have shown potential in diverse areas of drug discovery. We investigated the 3-hydroxy-4-pyridones **1a**–**1c** and 3-hydroxy-4-thiopyridones **1d**–**1f** as well as their Ru(η^6^-*p*-cymene)Cl complexes **2a**–**2f**, and report here the molecular structures of **1b** and **1d** as determined by X-ray diffraction analysis. Detailed cell biological investigations revealed potent cytotoxic activity, in particular of the 3-hydroxy-4-thiopyridones **1d**–**1f**, while the Ru complexes of both compound types were less potent, despite still showing antiproliferative activity in the low μM range. The compounds did not modulate the cell cycle distribution of cancer cells but were cytostatic in A549 and cytotoxic in NCI-H522 non-small lung cancer cells, among other effects on cancer cells.

## 1. Introduction

**Figure 1:**
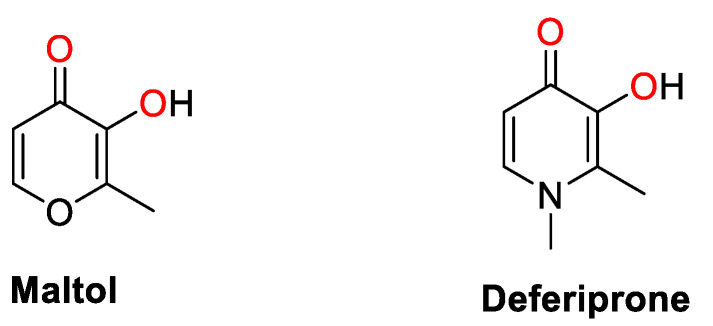
Chemical structures of maltol and deferiprone as representative examples of hydroxypyr(id)ones, and their chelating atoms highlighted in red.

Hydroxypyrones are a class of naturally occurring, biocompatible, heterocyclic ketones, with a hydroxyl group in the *ortho* or *para* position. Some examples, such as maltol, kojic acid, and their analogues (Figure 1), are commercially available and are used in the food and cosmetics industries [1,2,3,4]. Hydroxypyrones can be converted to hydroxypyridones (HPs) by substituting the endocyclic oxygen atom with a more nucleophilic nitrogen through the reaction with primary amines. HPs exhibit aromaticity and stronger chelation of metal ions than their hydroxypyrone analogs. HPs have attracted increasing interest due to their wide-ranging biomedical applications including as antibacterial [1,5,6] and antiviral agents [6], anthrax lethal factor inhibitors [1,6], magnetic resonance imaging contrast agents [7,8,9,10,11,12,13,14], matrix metalloprotein inhibitors [2,15,16], receptors for Li^+^ or Na^+^ [17,18,19], antidiabetic agents [20,21,22,23], and to restore disorders resulting from metal overload [24,25]. Most notably, deferiprone [1,2-dimethyl-3-hydroxy-4(1*H*)-one (Figure 1)] was the first orally administered drug to treat iron overload in thalassemia major, in particular for patients where deferoxamine therapy is ineffective [26,27]. It has also been shown that deferiprone has antiproliferative properties and can inhibit a subset of iron-dependent histone lysine demethylases. The lead derivatives based on deferiprone inhibited HP1-mediated gene silencing as well as the growth of breast tumors in murine xenograft models [28], but the exact mode of action is elusive. However, the depletion of intracellular iron and zinc as well as the inhibition of DNA synthesis via inactivation of ribonucleotide reductase are attributed as key contributing factors to their antiproliferative properties [29,30,31,32].

Thiopyr(id)ones (thio-HPs) can be prepared by thionation of the respective pyr(id)ones [33,34,35]. The thio-HPs form an important class of non-hydroxamate chemotypes that can chelate Zn^II^ ions. In this context, they have been studied as inhibitors of zinc-dependent metalloenzymes such as histone deacetylases (HDACs) and matrix metalloproteases (MMPs). Both HDACs and MMPs are important cellular targets that are involved in a variety of pathologies [15,33]. Oyelere and coworkers identified lead 3-hydroxypyridin-2-thiones that inhibited HDAC6 and HDAC8 with 50% inhibitory concentration (IC_50_) values in the nM range showing selectivity over HDAC1 [36]. The most potent HDAC inhibitors were cytotoxic against various cancer cell lines including J.*γ*1, a mutant Jurkat cell line resistant to the clinically used HDAC inhibitor suberanilohydroxamic acid (SAHA, vorinostat) and other 3-hydroxypyridin-2-thiones [37]. Cohen and coworkers carried out seminal work to explore the properties of HPs and thio-HPs as inhibitors of MMPs and found thio-HPs, in particular, were potent inhibitors of MMPs, which was attributed to the thiophilicity of the zinc(II) ion [15,33]. In this context, HPs are O,O-chelating ligands similar to hydroxamic acids and catechols. They can be easily deprotonated at the hydroxyl group and thereby act as bidentate anionic ligands [1,2,3,4]. Therefore, HPs are excellent metal chelators, and their coordination chemistry has been explored with a wide range of metals, including Ru(arene) compounds with antitumor properties [34,35,38,39,40,41]. Initial studies demonstrated low cytotoxicity due to the formation of the inert trishydroxido-bridged diruthenium hydrolysis product, which could be overcome by substituting HPs for thio-HPs, and the complexes were identified as significantly more antiproliferative [34,35,38,39,40,41]. Alternatively, we introduced ditopic ligands and their coordination to Ru(arene) moieties gave compounds with high in vitro anticancer activity [42,43].

In our continued research efforts to design new anticancer agents, we aimed to further explore the tumor-inhibiting properties of hydroxy(thio)pyridines and their Ru complexes [33,35,44], by examining the impact of the different chelating groups and of the substituent on the endocyclic nitrogen atom on the biological activity, as well as their organoruthenium complexes.

## 2. Experimental Section

### 2.1. Materials and Methods

All air-sensitive reactions were performed under nitrogen in standard Schlenk flasks. 3-Hydroxy-2-methyl-4(*1H*)-pyrone (maltol), Lawesson’s reagent, benzylamine, ethylbenzylamine, and 4-methylbenzylamine were purchased from Sigma Aldrich; ruthenium(III) chloride hydrate (99%), from Precious Metals Online; and sodium methoxide from Fluka. Dichloromethane, diethyl ether, and tetrahydrofuran were dried through a solvent purification system (LC Technology Solutions Inc., SP-1 solvent purifier) and transferred into Schlenk flasks that were dried under vacuum and degassed with N_2_ prior to use. Ethanol and methanol were dried over activated molecular sieves (3 Å) in a N_2_ Erlenmeyer flask for two days prior to use.

The dimeric precursor bis[dichlorido(η^6^-p-cymene)ruthenium(II)] [45], 1-benzyl-2-methyl-3-hydroxypyridin-4(1*H*)-one (**1a**) [33,35], 1-ethylbenzyl-2-methyl-3-hydroxypyridin-4(1*H*)-one (**1b**) [44], and 1-(4-methylbenzyl)-2-methyl-3-hydroxypyridin-4(1*H*)-one (**1c**) [44] were prepared according to literature procedures. 1-Benzyl-2-methyl-3-hydroxypyridin-4(1*H*)-thione (**1d**) [33,35] and 1-ethylbenzyl-2-methyl-3-hydroxypyridin-4(1*H*)-thione (**1e**) [33] were reported earlier, however, using different procedures.

Silica gel (60 F254 Merck) -coated aluminum sheets were used for thin-layer chromatography. The ^1^H and ^13^C{^1^H} nuclear magnetic resonance (NMR) spectra were collected on a Bruker Avance spectrometer AVIII 400 MHz at 400.13 MHz (^1^H) and 100.61 (^13^C{^1^H}) at ambient temperature (see Figures S7–S21 for the ^1^H and ^13^C{^1^H} NMR spectra). For NMR experiments, DMSO-*d*_6_, MeOH-*d*_4_, and CDCl_3_ were used as solvents and residual solvent peaks were used as reference for peak assignments. Mass spectra were obtained on a Bruker micrOTOF-Q II mass spectrometer in positive and negative ion electrospray ionization (ESI) modes. Elemental analyses were carried out on an Exeter Analytical Inc-CE-440 Elemental Analyser.

The X-ray diffraction data sets of **1b** and **1d** were collected on a Bruker Smart APEX II diffractometer with graphite-monochromatized Mo*Kα* radiation, λ_Mo_ = 0.71073 Å at 100 K (Table S1). Data reduction was carried out using the SAINT program [46]. Semi-empirical absorption corrections were applied based on equivalent reflections using SADABS [47]. The structure solution and refinements were performed with the SHELXS-97 and SHELXL-2013 program packages [48].

### 2.2. Syntheses

#### 2.2.1. General Procedure for the Synthesis of 1-Substituted 2-Methyl-3-hydroxypyridin-4-(1H)-thiones (**1d**–**1f**)

A solution of **1a**–**1c** (1 equiv.) and Lawesson’s reagent (1.5 equiv.) in tetrahydrofuran was heated at 60 °C for 4–6 h. After the completion of the reaction, the reaction mixture was cooled and concentrated under reduced pressure. The pure product was isolated by column chromatography on silica gel using dichloromethane as the eluent. A solution of the product in methanol (MeOH)/dichloromethane (DCM) was kept at 4 °C. After 12 h, needle-like crystals formed, which were filtered, washed with cold methanol, and dried under vacuum.

#### 2.2.2. 1-Benzyl-2-methyl-3-hydroxypyridin-4(1H)-thione, **1d**

Compound **1d** was synthesized according to the general procedure using **1a** (2.15 g, 10 mmol) and Lawesson’s reagent (6.0 g, 15 mmol). Yield: 61% (1.42 g), MS (ESI^+^); *m/z* = 232.0783 [M + H]^+^. Anal. found: C, 66.04; H, 6.01; N, 5.62; Calc. for C_13_H_13_NOS·0.25H_2_O: C, 66.21; H, 5.77; N, 5.94. ^1^H NMR (400.13 MHz, DMSO-*d*_6,_ 25 °C): *δ* = 2.31 (s, 3H, H-7), 5.46 (s, 2H, H-N), 6.94–7.85 (m, 7H, H-5, H-Ph, H-6) ppm.

#### 2.2.3. 1-Ethylbenzyl-2-methyl-3-hydroxypyridin-4(1H)-thione, **1e**

Compound **1e** was synthesized according to the general procedure using **1b** (2.45 g, 10 mmol) and Lawesson’s reagent (6.0 g, 15 mmol). Yield: 60% (1.48 g), MS (ESI^+^); *m/z* = 268.0767 [M + Na]^+^. Anal. found: C, 66.04; H, 6.01; N, 5.62; Calc. for C_14_H_15_NOS·0.5H_2_O: C, 66.11; H, 6.34; N, 5.51. ^1^H NMR (400.13 MHz, DMSO-*d*_6_, 25 °C): *δ* = 2.41 (s, 3H, H-7), 3.05 (t, 2H, H-Ph, ^3^*J* = 7 Hz), 4.36 (t, 2H, H-N, ^3^*J* = 7 Hz), 7.21–7.32 (m, 6H, H-5, H-Ph), 7.54 (d, 1H, H-6, ^3^*J* = 7 Hz) ppm.

#### 2.2.4. 1-(p-Methylbenzyl)-2-methyl-3-hydroxypyridin-4(1H)-thione, **1f**

Compound **1f** was synthesized according to the general procedure using **1c** (2.45 g, 10 mmol) and Lawesson’s reagent (6.0 g, 15 mmol). Yield: 65% (1.59 g), MS (ESI^+^); *m/z* = 268.0771 [M + Na]^+^. Anal. found: C, 60.09; H, 6.43; N, 5.22; Calc. for C_14_H_15_NOS·2H_2_O: C, 59.75; H, 6.81; N, 4.98. ^1^H NMR (400.13 MHz, MeOH-*d*_4_, 25 °C): *δ* = 2.01 (s, 3H, H-Ph), 2.39 (s, 3H, H-7), 5.42 (s, 2H, H-N), 7.11–7.15 (m, 2H, H-Ph), 7.36–7.40 (m, 2H, H-Ph), 7.47 (d, 1H, H-5, ^3^*J* = 7 Hz), 7.65 (d, 1H, H-6, ^3^*J* = 7 Hz) ppm.

### 2.3. General Procedure for the Synthesis of Ru(η^6^-p-Cymene) Complexes (***2a**–**2f***)

Complexes **2a**–**2f** were prepared following the literature procedure for related compounds [34,35,40]. One equiv. of **1a**–**1f** was stirred with 1.2 equiv. of NaOMe for about 30 min in methanol. Then, 0.5 equiv. bis[dichlorido(η^6^-p-cymene)ruthenium(II)] was added as a solid in one portion. The reaction mixture was stirred for 4–5 h, then the solvent was evaporated under reduced pressure. The residue was dissolved in dichloromethane and filtered to remove salts. The products **2a**–**2f** were precipitated by addition of hexane to the dichloromethane solution of the complexes.

#### 2.3.1. [Chlorido{3-oxo-1-benzyl-2-methylpyridin-4(1H)-onato-κ^2^O,O}(η^6^-p-cymene)ruthenium(II)], **2a**

Complex **2a** was synthesized from bis[dichlorido(η^6^-p-cymene)ruthenium(II)] (171 mg, 0.28 mmol), **1a** (120 mg, 0.56 mmol), and sodium methoxide (NaOMe) (24 mg, 0.67 mmol). Yield: 36% (108 mg), MS (ESI^+^); *m*/*z* = 450.1013 [M − Cl]^+^ (calcd. 450.1008). Anal. found: C, 52.63; H, 4.86; N, 2.70; Calc. for C_23_H_26_ClNO_2_Ru·0.6CH_2_Cl_2_: C, 52.89; H, 5.12; N, 2.61. ^1^H NMR (400.13 MHz, MeOH-*d*_4_, 25 °C): *δ* = 1.32 (d, 6H, H-15/H-17, ^3^*J* = 7 Hz), 2.25 (s, 3H, H-7), 2.33 (s, 3H, H-14), 2.81–2.87 (m, 1H, H-16), 5.32 (s, 2H, N-CH_2_), 5.42 (d, 2H, H-9/H-13, ^3^*J* = 6 Hz), 5.65 (d, 2H, H-10/H-12, ^3^*J* = 6 Hz), 6.57 (d, 1H, H-5,), 7.01 (d, 2H, Ph, ^3^*J* = 7 Hz), 7.28–7.37 (m, 3H, Ph), 7.55 (d, 1H, H-6, ^3^*J* = 7 Hz) ppm. ^13^C{^1^H} NMR (100.61 MHz, MeOH-*d*_4_, 25 °C): *δ* = 12.1 (C-7), 18.4 (C-15,C-17), 22.6 (C-14), 32.4 (C-16), 59.1 (C-Ph), 78.9 (C-9/C-13), 80.6 (C-10/C-12), 97.0 (C-11), 100.0 (C-8), 110.4 (C-5), 127.4 (C-Ph), 129.3 (C-Ph), 130.2 (C-Ph), 135.7 (C-6), 135.8 (C-2), 137.0 (C-Ph), 160.9 (C-3), 175.7 (C-4) ppm.

#### 2.3.2. [Chlorido{3-oxo-1-ethylbenzyl-2-methylpyridin-4(1H)-onato-κ^2^O,O}(η^6^-p-cymene)ruthenium(II)], **2b**

Complex **2b** was synthesized from bis[dichlorido(η^6^-p-cymene)ruthenium(II)] (159 mg, 0.26 mmol, **1b** (119 mg, 0.52 mmol), and NaOMe (34 mg, 0.63 mmol). Yield: 37% (104 mg), MS (ESI^+^); *m/z* = 464.1159 [M − Cl]^+^ (calcd. 464.1165). Anal. found: C, 56.41; H, 5.36; N, 3.10; Calc. for C_24_H_28_ClNO_2_Ru·0.5H_2_O: C, 56.74; H, 5.75; N, 2.76. ^1^H NMR (400.13 MHz, CDCl_3_, 25 °C): *δ* = 1.28 (d, 3H, H-15/H-17, ^3^*J* = 7 Hz), 1.32 (d, 3H, H-15/H-17, ^3^*J* = 7 Hz), 2.31 (s, 3H, H-7), 2.43 (s, H-14), 2.91 (m, 1H, H-16), 2.95 (t, 2H, Ph-CH_2_, ^3^*J* = 7 Hz), 4.02 (q, 2H, N-CH_2_, ^3^*J* = 7 Hz, ^2^*J* = 16 Hz), 5.24 (d, 1H, H-9/H-13, ^3^*J* = 6 Hz), 5.29 (d, 1H, H-9/H-13, ^3^*J* = 6 Hz), 5.43 (d, 1H, H-10/H-12, ^3^*J* = 6 Hz), 5.49 (d, 1H, H-10/H-12, ^3^*J* = 6 Hz), 6.32 (d, 1H, H-5, ^3^*J* = 7 Hz), 6.70 (d, 1H, H-6, ^3^*J* = 7 Hz), 7.00–7.03 (m, 2H, Ph), 7.23–7.29 (m, 3H, Ph) ppm. ^13^C{^1^H} NMR (100.61 MHz, CDCl_3_, 25 °C): *δ* = 11.8 (C-7), 18.6 (C-15, C-17), 22.4 (C-14), 31.0 (C-16), 37.1 (C-Ph), 55.9 (C-N), 77.6, 78.6, (C-9/C-13), 79.2, 79.5 (C-10/C-12), 95.4 (C-11), 99.0 (C-8), 109.3 (C-5), 127.2, 128.7, 128.9 (C-Ph), 131.4 (C-6), 131.5 (C-2), 136.4 (C-Ph), 161.1 (C-3), 175.3 (C-4) ppm.

#### 2.3.3. [Chlorido{3-oxo-1-(p-methylbenzyl)-2-methylpyridin-4(1H)-onato-κ^2^O,O}(η^6^-p-cymene)ruthenium(II)], **2c**

Complex **2c** was synthesized from bis[dichlorido(η^6^-p-cymene)ruthenium(II)] (159 mg, 0.26 mmol), **1c** (119 mg, 0.52 mmol), and NaOMe (34 mg, 0.63 mmol). Yield: 32% (97 mg), MS (ESI^+^); *m/z* = 464.1162 [M − Cl]^+^ (calcd. 464.1165). Anal. found: C, 51.67; H, 5.07; N, 2.54; Calc. for C_24_H_28_ClNO_2_Ru·CH_2_Cl_2_: C, 51.42; H, 5.18; N, 2.40. ^1^H NMR (400.13 MHz, MeOH-*d*_4_, 25 °C): *δ* = 1.30 (d, 6H, H-15/H-17, ^3^*J* = 7 Hz), 2.24 (s, 3H, H-7), 2.30 (s, 3H, H-14), 2.31 (s, 3H, Ph-CH_3_), 2.80–2.87 (m, 1H, H-16), 5.23 (s, 2H, N-CH_2_), 5.37 (d, 2H, H-9/H-13, ^3^*J* = 6 Hz), 5.60 (d, 2H, H-10/H-12, ^3^*J* = 6 Hz), 6.51 (d, 1H, H-5, ^3^*J* = 7 Hz), 6.91 (d, 2H, Ph, ^3^*J* = 8 Hz), 7.15 (d, 2H, Ph, ^3^*J* = 8 Hz), 7.48 (d, 1H, H-6, ^3^*J* = 7 Hz) ppm. ^13^C{^1^H} NMR (100.61 MHz, MeOH-*d*_4_, 25 °C): δ = 12.1 (C-7), 18.6 (C-15,C-17), 21.1 (C-Ph), 22.6 (C-16), 32.3 (C-14), 59.0 (C-N), 79.0 (C-9/C-13), 80.7 (C-10/C-12), 96.9 (C-11), 100.0 (C-8), 110.3 (C-5), 127.4, 130.7 (C-Ph), 134.0 (C-2), 135.5 (C-6), 135.7 (C-Ph), 139.3 (C-Ph), 160.9 (C-3), 175.6 (C-4) ppm.

#### 2.3.4. [Chlorido{3-oxo-1-benzyl-2-methylpyridin-4(1H)-thionato-κ^2^O,S}(η^6^-p-cymene)ruthenium(II)], **2d**

Complex **2d** was synthesized from bis[dichlorido(η^6^-p-cymene)ruthenium(II)] (171 mg, 0.28 mmol), **1d** (130 mg, 0.56 mmol), and NaOMe (36 mg, 0.67 mmol). Yield: 62% (186 mg), MS (ESI^+^); *m*/*z* = 466.0780 [M − Cl]^+^ (calcd. 466.0779). Anal. found: C, 52.61; H, 5.34; N, 2.54; Calc. for C_23_H_26_ClNOSRu·0.4CH_2_Cl_2_: C, 52.53; H, 5.05; N, 2.62. ^1^H NMR (400.13 MHz, CDCl_3_, 25 °C): *δ* = 1.32 (d, 6H, H-15/H-17, ^3^*J* = 7 Hz), 2.25 (s, 3H, H-7), 2.33 (s, 3H, H-14), 2.81–2.87 (m, 1H, H-16), 5.32 (s, 2H, N-CH_2_), 5.42 (d, 2H, H-9/H-13, ^3^*J* = 6 Hz), 5.65 (d, 2H, H-10/H-12, ^3^*J* = 6 Hz), 6.57 (d, 1H, H-5, ^3^*J* = 7 Hz), 7.01 (d, 2H, Ph, ^3^*J* = 7 Hz), 7.28–7.37 (m, 3H, Ph), 7.55 (d, 1H, H-6, ^3^*J* = 7 Hz) ppm. ^13^C{^1^H} NMR (100.61 MHz, CDCl_3_, 25 °C): *δ* = 12.1 (C-7), 18.4 (C-15/C-17), 22.6 (C-14), 32.4 (C-16), 59.1 (C-N), 78.9 (C-9/C-13), 80.6 (C-10/C12), 97.0 (C-11), 100.0 (C-8), 110.4 (C-5), 127.4,129.3, 130.2 (C-Ph), 135.7 (C-6), 135.8 (C-2), 137.0 (C-Ph), 160.9 (C-3), 175.7 (C-4) ppm.

#### 2.3.5. [Chlorido{3-oxo-1-ethylbenzyl-2-methylpyridin-4(1H)-thionato-κ^2^O,S}(η^6^-p-cymene)ruthenium(II)], **2e**

Complex **2e** was synthesized from bis[dichlorido(η^6^-p-cymene)ruthenium(II)] (159 mg, 0.26 mmol), **1e** (127 mg, 0.52 mmol), and NaOMe (34 mg, 0.63 mmol). Yield: 51% (154 mg), MS (ESI^+^); *m/z* = 480.0936 [M − Cl]^+^ (calcd. 480. 0935). Anal. found: C, 51.47; H, 5.31; N, 2.40; Calc. for C_24_H_28_ClNOSRu·0.7CH_2_Cl_2_: C, 51.64; H, 5.16; N, 2.44. ^1^H NMR (400.13 MHz, CDCl_3_, 25 °C): *δ =* 1.27 (d, 6H, H-15/H-17, ^3^*J* = 7 Hz), 2.21 (s, 3H, H-7), 2.47 (s, H-14), 2.76–2.83 (m, 1H, H-16), 2.98 (t, 2H, Ph-CH_2_, ^3^*J* = 7 Hz), 4.12 (t, 2H, N-CH_2_, ^3^*J* = 7 Hz), 5.22 (d, 2H, H-9/H-13, ^3^*J* = 6 Hz), 5.46 (d, 2H, H-10/H-12, ^3^*J* = 6 Hz), 6.61 (d, 1H, H-5, ^3^*J* = 6 Hz), 7.03–7.06 (m, 3H, H-6, Ph), 7.25–7.29 (m, 3H, Ph) ppm. ^13^C{^1^H} NMR (100.61 MHz, CDCl_3,_ 25 °C): *δ* = 12.4 (C-7), 18.5 (C-15/C-17), 22.7 (C-14), 31.1 (C-16), 37.0 (C-Ph), 57.0 (C-Ph), 80.6 (C-9/C-13), 82.7 (C-10/C-12), 97.2 (C-8), 99.3 (C-11), 121.5 (C-5), 126.0 (C-Ph), 127.5 (C-6), 128.8 (C-Ph), 129.1 (C-Ph), 132.9 (C-2), 136.2 (C-Ph), 163.8 (C-3), 168.3 (C-4) ppm.

#### 2.3.6. [Chlorido{3-oxo-1-(p-methylbenzyl)-2-methylpyridin-4(1H)-thionato-κ^2^O,S}(η^6^-p-cymene)ruthenium(II)], **2f**

Complex **2f** was synthesized from bis[dichlorido(η^6^-p-cymene)ruthenium(II)] (159 mg, 0.26 mmol), **1f** (127 mg, 0.52 mmol), and NaOMe (34 mg, 0.63 mmol). Yield: 61% (177 mg), MS (ESI^+^); *m/z* = 480.0941 [M − Cl]^+^ (calcd. 480.0935). Anal. found: C, 52.47; H, 5.29; N, 2.38; Calc. for C_24_H_28_ClNOSRu·0.5CH_2_Cl_2_: C, 52.78; H, 5.24; N, 2.51. ^1^H NMR (400.13 MHz, CDCl_3_, 25 °C): *δ =* 1.25 (d, 6H, H-15/H-17, ^3^*J* = 7 Hz), 2.19 (s, 3H, H-7), 2.32 (s, 3H, Ph-CH_3_), 2.39 (s, 3H, H-14), 2.76–2.83 (m, 1H, H-16), 5.07 (s, 2H, N-CH_2_), 5.20 (d, 2H, H-9/H-13, ^3^*J* = 6 Hz), 5.45 (d, 2H, H-10/H-12, ^3^*J* = 6 Hz), 6.85 (d, 1H, H-5, ^3^*J* = 6 Hz), 6.89 (d, 2H, Ph, ^3^*J* = 8 Hz), 7.10–7.16 (m, 3H, H-6, Ph) ppm. ^13^C{^1^H} NMR (100.61 MHz, CDCl_3_, 25 °C): δ = 12.7 (C-7), 18.5 (C-15/C-17), 21.2 (CH_3_-Ph), 22.7 (C-14), 31.1 (C-16), 58.8 (C-Ph), 80.5 (C-9/C-13), 82.8 (C-10/C-12), 97.3 (C-11), 99.5 (C-8), 121.7 (C-5), 126.5 (C-6), 126.6 (C-Ph), 130.0 (C-Ph), 131.0 (C-2), 133.8 (C-Ph), 138.7 (C-Ph), 164.4 (C-3), 168.5 (C-4) ppm.

### 2.4. Antiproliferative Activity Studies

#### 2.4.1. Cell Maintenance

SiHa cells were from Dr. David Cowan, Ontario Cancer Institute, Canada, while all the other cancer cells were supplied by American Type Culture Collection. A549, NCI-H522 lung cancer, and PNT1A normal prostate epithelial cell lines were seeded in Roswell Park Memorial Institute (RPMI) 1640 media supplemented with 2%, 10%, and 10% fetal bovine serum (FBS; Moregate Biotech, Hamilton, New Zealand), respectively, and 1% penicillin–streptomycin (PenS) purchased from ThermoFisher Scientific (Waltham, MA, USA). MDA-MB-231, MDA-MB-468, and PC3 cells were grown in Dulbecco’s Modified Essential Medium (DMEM) and Ham’s F-12 (DMEM/F12; Life Technologies) media supplemented with 10%, 10%, and 5% FBS, and 1% PenS. HCT116, SW480, SiHa, and NCI-H460 were grown in Minimum Essential Medium α (αMEM; ThermoFisher Scientific, Waltham, MA, USA) supplemented with 5% FBS. All cells were cultured at 37 °C in 5% CO_2_ and 95% humidified air.

#### 2.4.2. Drug Preparation

Stock solutions of **1a**–**1f** and SAHA (AK Scientific, Union City, CA, USA) were prepared by dissolving in 100% DMSO and stored at −20 °C. Since the metal-based drugs could interact with DMSO in long-term storage, all the ruthenium complexes **2a**–**2f** were freshly prepared by dissolving in 100% DMSO. The stocks of all the drugs were diluted into serum-free media to a final concentration of 0.5% DMSO. DMSO (0.5%) was used as the vehicle control.

#### 2.4.3. Sulforhodamine B (SRB) Cytotoxicity Assay

The cells were seeded at 750 (HCT116, NCI-H460), 4000 (SiHa, PC3, A549), 5000 (SW480), 8000 (MDA-MB-231), 10,000 (NCI-H522 and PNT1A), and 15,000 (MDA-MB-468) cells/well in 96-well plates and left to attach for 24 h. The compounds were added to the plates in a series of 3-fold dilutions, containing a maximum of 0.5% DMSO at the highest concentration. At the end of the 72-h incubation period, the viable cell number in each well was determined by the SRB assay [49]. The half-maximal inhibitory concentration (IC_50_) or half-maximal effective concentration (EC_50_) values were calculated with SigmaPlot 12.5 (Systat Software, San Jose, CA, USA) or Prism-GraphPad 8 software (San Diego, CA, USA) using a three-parameter logistic sigmoidal dose−response curve between the calculated growth inhibition and the compound concentration. The presented EC_50_ values are the mean of at least three independent experiments, where 10 concentrations were tested in duplicate for each compound. The selectivity index (SI) of **1d** and **1e** was calculated according to Equation (1) [50]:

(1) $$\mathrm{SI}=\frac{{\mathrm{EC}}_{50}\;\mathrm{of}\;\mathrm{drug}\;\mathrm{in}\;\mathrm{PNT}1A\;\mathrm{cells}}{{\mathrm{EC}}_{50}\;\mathrm{of}\;\mathrm{drug}\;\mathrm{in}\;\mathrm{cancer}\;\mathrm{cell}\;\mathrm{line}}$$

Time course cytotoxicity studies were performed by treating A549 and NCI-H522 cells (4000 and 10,000 cells, respectively) with 2× the EC_50_ concentration of **1d** and **1e** for 12 to 72 h and the cell number was determined via the SRB assay. The same concentrations of **1d** and **1e** were used to investigate the effect of 72 h of drug withdrawal on the growth of A549 and NCI-H522 cells.

### 2.5. Clonogenic Assay

A549 and NCI-H522 cells (1.0 × 10^6^ and 2.5 × 10^6^ cells, respectively) were seeded in 10-cm cell culture dishes and incubated for 24 h in 5% CO_2_ at 37 °C. Cells were then treated with **1d** and **1e** at 2× the EC_50_ concentrations while vehicle control cells were treated with DMSO (0.5%). At the end of the 72-h treatment period, A549 and NCI-H522 cells (2 × 10^2^ and 5 × 10^2^ cells, respectively) were seeded in 6-well plates for 14 and 21 days, respectively, and the medium was replaced every three days. At the end of the incubation period, the cells were fixed in methanol, followed by a sterile phosphate-buffered saline (PBS) wash, and stained with crystal violet dye for 15 min. Each well was then washed with water and left to dry overnight [51]. The number of colonies containing more than 50 cells was determined using Evos Xl Core Cell Imaging System, Thermo Fisher Scientific (Waltham, MA, USA).

### 2.6. Cell Cycle Analysis

A549 (1.0 × 10^6^ cells per dish) and NCI-H522 cells (3.0 × 10^5^ cells per well) were seeded in 10-cm cell culture dishes and incubated for 24 h. The cells were then treated with **1d** or **1e** at 1× and 2× the EC_50_ value or DMSO (0.5 %) for 6 and 12 h. The cells were then harvested, washed with PBS, and fixed in ice-cold 70% ethanol. Following rehydration with PBS, the samples were centrifuged and the cell pellets were suspended in FxCycle propidium iodide (PI)/RNase staining solution (Life Technologies Corporation, Carlsbad, CA, USA) and incubated in the dark for 30 min at room temperature [52]. The samples were then analyzed with a Gallios BD flow cytometer and the data were analyzed with FlowJo LLC software. Data are presented as the mean proportion of cells in each phase ± standard error of the mean (SEM) obtained from three independent experiments.

### 2.7. Apoptosis Assay

NCI-H522 cells (3.0 × 10^5^ cells per well) were seeded in 6-well plates and incubated for 24 h. The cells were then treated with **1d** or **1e** at 1× and 2× the EC_50_ value or DMSO (0.5%) for 12 and 24 h. The cells were then washed with PBS, trypsinized, and centrifuged to collect the cell pellet. The cell pellet was suspended in 100 µL of binding buffer (10 mM HEPES, 140 mM NaCl, 2.5 mM CaCl_2_). Annexin-V-FLUOS/PI (Roche Diagnostics Corp.) and 100 µL of binding buffer was added to each sample and incubated for 30 min in the dark at room temperature [52,53]. The samples were then analyzed on Gallios BD flow cytometer. The data were analyzed using Kaluza Analysis Software. Three independent experiments were conducted and the results are presented as the number of apoptotic cells as a percent of total cell number.

### 2.8. HDAC Inhibition

HDAC1 and HDAC8 inhibition assays were performed using a fluorescent HDAC activity assay kit (Reaction Biology CORP., Malvern, PA, USA). The substrates were the fluorogenic peptides RHK_Ac_K_Ac_AMC (for HDAC8; residues 379–382 from p53) and RHKK_Ac_AMC (for HDAC1 and 6; residues 379–382 from p53). Initially, the inhibition of HDAC8 by **1a**, **1b**, **1d**, **1e**, **2a**, **2b**, **2d**, and **2e** was determined at a concentration of 10 μM (*n* = 2). The pyridones **1a** and **1b** and their complexes **2a** and **2b** were found to be inactive at this concentration and were not further evaluated. In contrast, the thiopyridones **1d** and **1e** and their Ru complexes **2d** and **2e** showed moderate inhibition of HDAC8 at 10 μM and were, therefore, further studied to determine their effect on acetylated histone-H3 by Western blotting.

### 2.9. Western Blotting

A549 (1.0 × 10^6^ cells per dish) and NCI-H522 cells (2.5 × 10^6^ cells per dish) were seeded in 10-cm cell culture dishes and incubated for 24 h. The cells were then treated with **1d** or **1e** at 1× and 2× the EC_50_ value or DMSO (0.5%) for 12 and 24 h. The cells were then lysed with lysis buffer (50 mM Tris base (pH 7.5), 150 mM NaCl, 1 mM EDTA, 1 mM EGTA, 0.5% NP-40, 0.5% SDS, 1 mM sodium orthovanadate, 2.5 mM sodium pyrophosphate, 10 mM sodium fluoride, 1 mM PMSF, and complete protease and phosphatase inhibitor cocktail). Cell lysates were then collected, sonicated, and centrifuged at 14,000 rpm for 8 min at 4 °C. Cell lysates were then boiled in Laemmli buffer and resolved by SDS-PAGE (25 µg/lane for A549 cells and 20 µg/lane for NCI-H522 cells) and were then transferred to a polyvinylidene fluoride (PVDF) membrane (Bio-Rad Laboratories, Hercules, CA, USA). Protein levels were analyzed with either acetylated histone-H3, cyclin D1 or cyclin B1, primary antibodies (Cell Signaling Technology, Danvers, MA, USA) followed by horseradish peroxide-conjugated secondary antibodies (Merck Millipore, Burlington, MA, USA). The film was scanned using a GS710 densitometer (Bio-Rad Laboratories). The protein density was calculated as a percentage of β-actin or β-tubulin (Cell Signaling Technology, Danvers, MA, USA). The results presented are from three independent experiments performed in triplicate.

### 2.10. Data Analysis

Cell viability and clonogenic assay results were analyzed with a one-way ANOVA coupled with a Bonferroni post-hoc test. Time course cytotoxicity, drug withdrawal effect, cell cycle, Western blotting, and apoptosis data were analyzed using a two-way ANOVA coupled with a Bonferroni post hoc test. All the statistical analyses were performed using Prism-GraphPad 8 software (USA). Data are expressed as the mean ± SEM. *p* < 0.01 was the minimal requirement for consideration of statistical significance.

## 3. Results and Discussion

### 3.1. Synthesis and Characterization

**Scheme 1:**
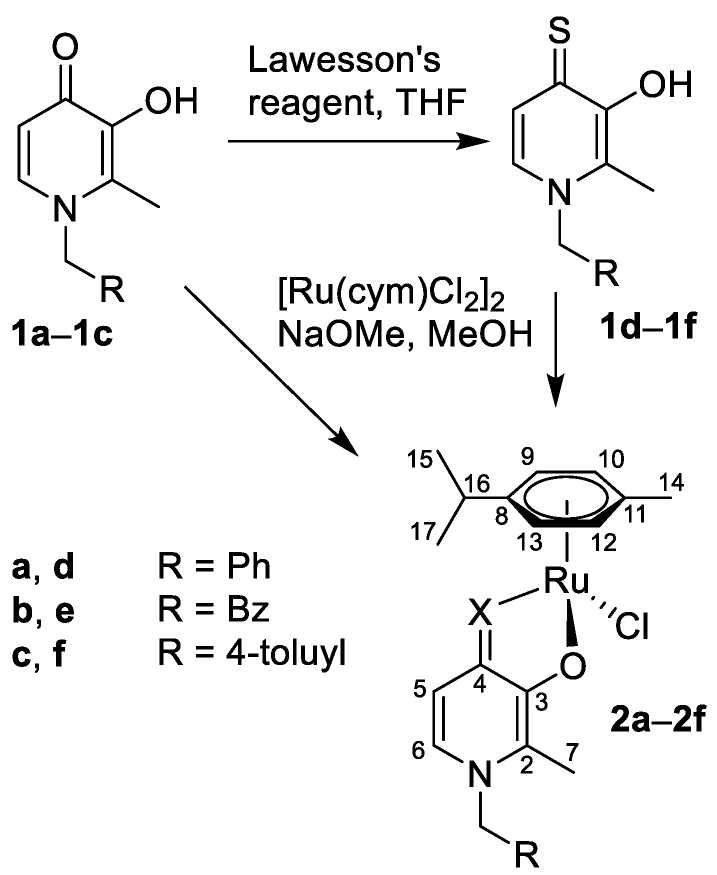
Synthesis of the investigated pyridones **1a**–**1c**, thiopyridones **1d**–**1f,** and the respective Ru(cym)Cl complexes **2a**–**2f**, and the NMR numbering scheme.

The preparation of the HPs and thio-HPs **1a**–**1f** was reported earlier using different methods [33,35,44]. We adapted the methods and obtained **1a**–**1c** (Scheme 1) by refluxing benzyl-protected maltol with the respective amines, i.e., benzylamine **a**, 2-phenylethan-amine **b**, and 4-methylbenzylamine **c**, in an ethanol/water solution [34,35,40,44]. The subsequent hydrogenolysis in methanol catalyzed by 10% Pd/C gave the 3-hydroxy-pyr(id)ones **1b** and **1c**, while the reaction with **1a** resulted in cleavage of the benzyl group from both positions 1 (-NBn) and 3 (-OBn). Therefore, **1a** was prepared following an alternative route, where maltol was reacted directly with benzylamine in 100% acetic acid to obtain **1a** in good yield (60%). To compare the cytotoxicity of HPs to those of thio-HPs, as well as their Ru complexes, **1a**–**1c** were converted to the respective thiopyridones **1d**–**1f** by heating them with Lawesson’s reagent in dry tetrahydrofuran at 60 °C for 4–6 h (Scheme 1). The crude product was purified by column chromatography on silica gel with dichloromethane as the eluent. The crystallization from a methanol/dichloromethane mixture gave pure products in good yields. The identity and purity of **1a**–**1f** was demonstrated by NMR spectroscopy, elemental analysis, and electrospray ionization mass spectrometry (ESI-MS).

**Figure 2:**
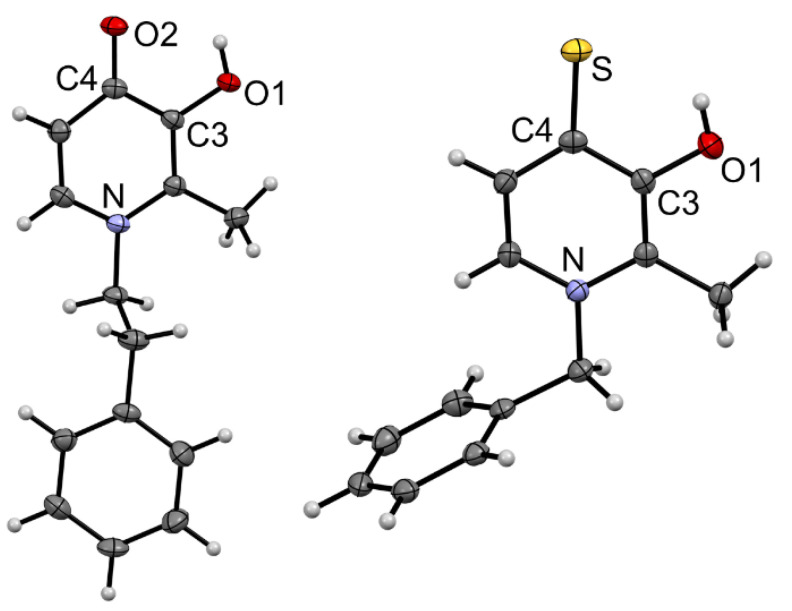
Molecular structures of **1b**·MeOH and **1d** drawn at 50% probability level. Solvent molecules have been omitted for clarity.

Single crystals of **1b** and **1d** were obtained by slow evaporation of a MeOH/dichloromethane solution (Figure 2). When comparing the bond lengths, as expected, the C4=O bond in **1b** was significantly shorter than the equivalent C4=S bond in **1d** (Table S2), as well as of the C3–O1 bonds with single-bond character. The C3–O1 bonds were minimally influenced by the substitution of the O_=C4_ for S_=C4_ and virtually identical bond lengths were detected. As expected, the atoms O2/S–C4–C3–O2 formed a plane, with the bonds from C3 to the adjacent carbon atoms having single- (C3–C4) and double-bond character (C2–C3). In both **1b** and **1d**, π stacking between the pyridone rings was observed (Figures S1 and S2). In the case of **1b**, in addition, a H bond network was seen involving both the OH and O_=C4_ groups as well as co-crystallized MeOH (Figure S1).

Both groups of pyridones and thiopyridones were converted into the respective Ru(cym)Cl (cym = η^6^-*p*-cymene) complexes **2a**–**2f** by deprotonating the 3-hydroxy-4-(thio)pyr(id)ones **1a**–**1f** with sodium methoxide in dry methanol, followed by the addition of [Ru(cym)Cl_2_]_2_ at ambient temperature and under inert atmosphere (Scheme 1), following a procedure that yielded **2d** and related compounds before [34,35,40,54,55]. After evaporation of methanol, the crude product was extracted with dichloromethane, filtered to remove inorganic salts, and pure **2a**–**2f** were precipitated from dichloromethane with *n*-hexane in good yields. The complexes were characterized by ^1^H and ^13^C{^1^H} NMR spectroscopy. Coordination of the bidentate *O*,*O*- and *O*,*S*-ligands to the Ru center resulted in downfield shifts for H-6 and H-5 in ^1^H NMR spectra, characteristic of successful complex formation. The ESI-mass spectra of the Ru compounds **2a**–**2f** featured the singly charged [M − Cl]^+^ cations as the base peaks. The theoretical isotope pattern was in good agreement with the experimentally obtained data.

### 3.2. Biological Properties

The cytotoxic activity of the (thio)pyridones **1a**–**1f** and their Ru complexes **2a**–**2f** was initially determined in human colorectal cancer (HCT116), non-small cell lung (NCI-H460), cervical (SiHa) and colon cancer (SW480) cell lines and is given for comparison purposes with our previous data as the IC_50_ value (Table 1). The thio-HP compounds **1d**–**1f** were found to be significantly more cytotoxic in all of the cancer cells investigated than their pyridone analogs **1a**–**1c**, with the thio-HP **1e** being the most cytotoxic derivative of all compounds investigated with IC_50_ values in the 50-nM range in HCT116 and SW480 cells. A similar trend was seen for the Ru complexes **2a**–**2f** with the thiopyridone complexes displaying IC_50_ values about an order of magnitude lower than the pyridone derivatives. However, complexation reduced the antiproliferative activity of both chelator types slightly and no selectivity for either of the cell lines could be identified. A similar trend was observed for **1d** and **2d** using the 3-(4,5-dimethylthiazol-2yl)-2,5-diphenyltetrazolium bromide (MTT) assay to investigate their cytotoxicity in A549, SW480, and CH1/PA-1 cells [35].

Due to these findings in the preliminary screen in cancer cells, we selected the most cytotoxic thiopyridones **1d** and **1e** and for comparison their Ru complexes **2d** and **2e** for investigations on their antiproliferative activity in a larger set of cell lines including lung (A549, NCI-H522), breast (MDA-MB-231, MDA-MB-468), and prostate (PC3) cancer cells. The trend observed in the preliminary screening was confirmed in this set of cell lines with the organic molecules **1d** and **1e** being more potent compared to the corresponding organometallics **2d** and **2e** (Table 2). Additionally, **1d** and **1e** displayed consistently higher cytotoxic activity (EC_50_ values of 0.23–0.36 µM) in the lung cancer cells compared to the other cell lines. To investigate the selectivity of **1d** and **1e**, the compounds were also screened on prostate epithelial cells (PNT1A). Both **1d** and **1e** displayed higher activity toward the lung cancer cells compared to both breast cancer cell lines. Compound **1d** was equally potent in prostate PC3 cancer cells (Table 2 and Table S3). Therefore, **1d** and **1e** were further examined in A549 and NCI-H522 non-small lung cancer (NSCLC) cells due to their more promising cytotoxic potential and higher selectivity in such cells.

**Figure 3:**
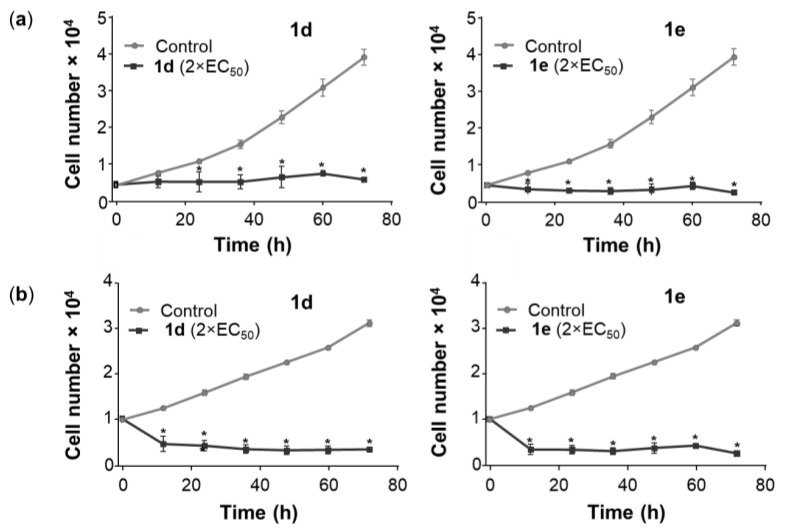
Time course cytotoxicity assessment of **1d** and **1e** in A549 and NCI-H522 cells. A549 cells and NCI-H522 were seeded in 96-well plates at 4000 and 10,000 cells per well, respectively, and incubated for 24 h at 37 °C. (**a**) A549 cells were treated with 0.72 µM of **1d** and 0.64 µM of **1e**, while (**b**) NCI-H522 cells were treated with 0.56 µM of **1d** and 0.46 µM of **1e**, in both cases for 12–72 h. Vehicle control cells were treated with DMSO (0.5%). At the end of the treatment period, the cell number was determined by the SRB assay. The points represent the mean ± SEM (*n* = 3). Data were analyzed using a two-way ANOVA coupled with a Bonferroni post hoc test. * Significantly different from control, *p* < 0.01. No significant differences were determined between each treatment point from 12 to 72 h.

To determine the cytotoxic activity of **1d** and **1e**, a time course cytotoxicity study was performed in A549 and NCI-H522 cells at 2× the EC_50_ concentration. Plotting the time against the cell number demonstrated that **1d** and **1e** showed almost identical cytostatic activity in A549 cells (Figure 3). In contrast, in NCI-H522 cells cytotoxic activity was observed under the same conditions (Figure 3).

**Figure 4:**
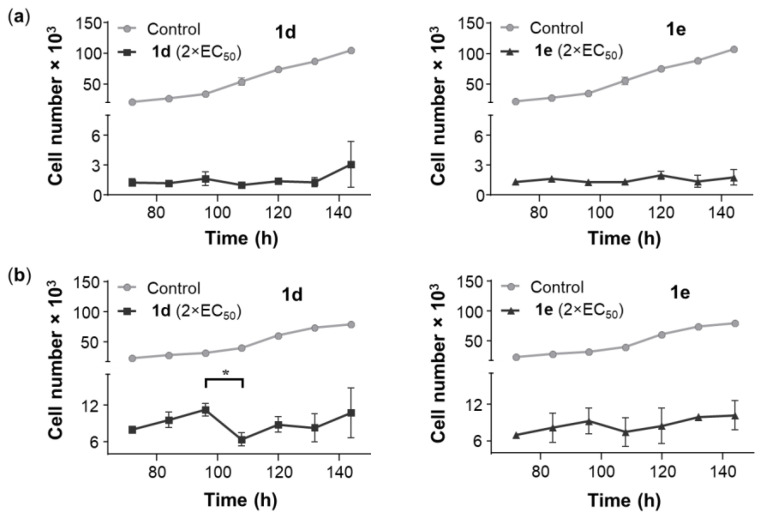
To study the drug withdrawal effect on A549 and NCI-H522 cell proliferation, (**a**) A549 cells (2000 cells/well) were treated with 0.72 µM of **1d** and 0.64 µM of **1e**, while (**b**) NCI-H522 cells (5000 cells/well) were treated with 0.56 µM of **1d** and 0.46 µM of **1e**, both for 72 h. Vehicle control cells were treated with DMSO (0.5%). Compound-containing medium was replaced with fresh medium, and the cells were allowed to grow for another 72 h. At the end of the treatment period, the cell number was determined by the SRB assay. The points represent the mean ± SEM (*n* = 3). Data were analyzed using a two-way ANOVA coupled with a Bonferroni post hoc test. * Indicates a significant difference between the data points, *p* < 0.01.

The majority of NSCLCs are incurable due to drug resistance, which leads to aggressive tumor variants [56,57]. To investigate the drug withdrawal effect of **1d** and **1e**, A549 and NCI-H522 cells were treated with **1d** and **1e** for 72 h and then cultured in the absence of drug for an additional 72 h. The number of viable A549 and NCI-H522 cells decreased significantly after 72-h exposure of either **1d** or **1e** but no significant changes in cell number were observed after drug withdrawal (Figure 4), suggesting that neither A549 nor NCI-H522 cells reverted to their typical growth pattern. Interestingly, the A549 cells did not regrow upon withdrawal of **1d** and **1e** despite the compounds being cytostatic (Figure 3a).

**Figure 5:**
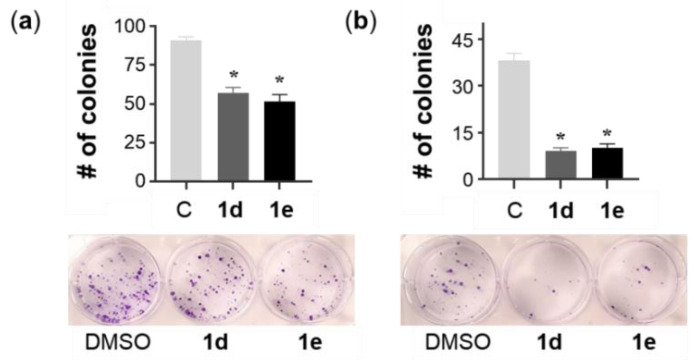
The effect of the withdrawal of **1d** and **1e** on the colony-formation ability of A549 and NCI-H522 cells. (**a**) A549 cells were treated with 0.72 µM of **1d** and 0.64 µM of **1e**, while (**b**) NCI-H522 cells were treated with 0.56 µM of **1d** and 0.46 µM of **1e**, each for 72 h. Vehicle control cells were treated with DMSO (0.5%). The A549 and NCI-H522 (2 × 10^2^ and 5 × 10^2^ cells, respectively) cells were then seeded in 6-well plates for 14 and 21 days, respectively, and the medium was changed every three days. Single-colony formation was determined by crystal violet staining. Bars represent the mean ± SEM (*n* = 3). Data were analyzed with a one-way ANOVA coupled with a Bonferroni post hoc test. * Indicates significant differences to control, *p* < 0.01.

To investigate the long-term efficacy of **1d** and **1e** on A549 and NCI-H522 single-cell growth, an in vitro clonogenic assay was performed. After 72 h of **1d** and **1e** exposure at 2× the EC_50_ concentration, A549 and NCI-H522 cells were cultured without drugs for 14 and 21 days, respectively. The results showed that the single-cell colony-formation ability of both cell lines decreased significantly compared to the control (Figure 5). The result of the clonogenic assay indicated that **1d** and **1e** were not able to prevent tumor relapse of A549 and NCI-H522 cells. This is in agreement with previous reports on the 2-pyridone pirfenidone (PFD), which was unable to suppress A549 and H4006 tumor cell relapse, whereas PFD-loaded liposomal formulations at the same concentration were highly effective [58].

**Figure 6:**
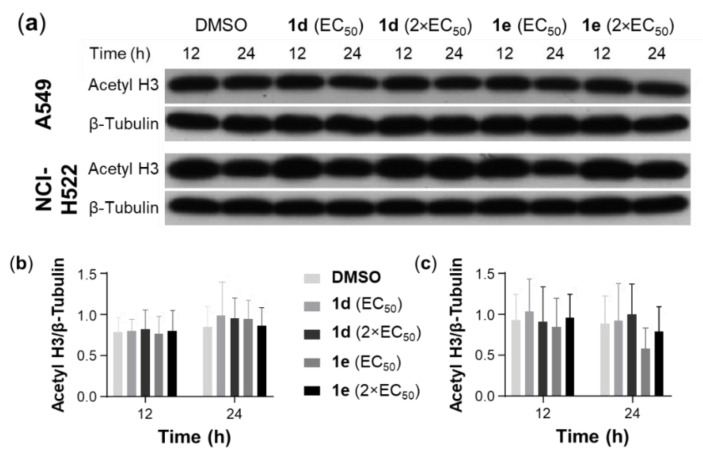
Effect of **1d** and **1e** on the expression of acetyl-H3 in A549 and NCI-H522 cells. (**a**) Cell lysates of A549 cells, which were treated with **1d** and **1e** at 1× and 2× the EC_50_ concentrations for 12 and 24 h, were subjected to Western blotting with a specific antibody against acetyl H3. Blots shown are representative of *n* = 3. Scanning densitometry of the ratio of acetyl-H3 to β-tubulin in (**b**) A549 and (**c**) NCI-H522 cells. Vehicle control cells were treated with DMSO (0.5%). Bars represent mean ± SEM (*n* = 3). Data were analyzed with a two-way ANOVA coupled with a Bonferroni post hoc test. No statistical significances were observed.

HDAC inhibitors attenuate acetylation events and block several cancer-related signaling pathways [59]. Small molecules containing zinc-binding groups, such as hydroxamic acids, have been among the most effective inhibitors of HDACs [60,61]. The 3-hydroxypyridin-2-thiones have shown potent and selective inhibitory activity for HDAC6 and HDAC8 with IC_50_ values in the nM range [36,37] and are structurally similar to **1d** and **1e**. Additionally, **1d** and **1e** were more potent compared to SAHA toward NSCLC cells investigated (Table 2 and Table 3). An initial screening for the inhibition of HDAC8 by **1a**, **1b**, **1d**, **1e**, **2a**, **2b**, **2d**, and **2e** at a concentration of 10 μM (*n* = 2) showed that the 3-hydroxy-pyridone derivatives **1a** and **1b** and their complexes **2a** and **2b** were inactive and consequently were not further evaluated. In contrast, the 3-hydroxypyridin-4-thione **1d** and **1e** and their Ru complexes **2d** and **2e** showed moderate inhibition of HDAC8 at 10 μM, compared to SAHA (Table 3). The HDAC inhibition properties of **1d** and **1e** were further investigated using Western blotting. The Western blots showed no significant difference in the expression of acetyl H3 in A549 and NCI-H522 cells after the treatment with either **1d** or **1e** at 1× and 2× the EC_50_ concentrations (Figure 6, Figures S4 and S5). These data suggest that HDAC8 is not a target for the compound type.

**Figure 7:**
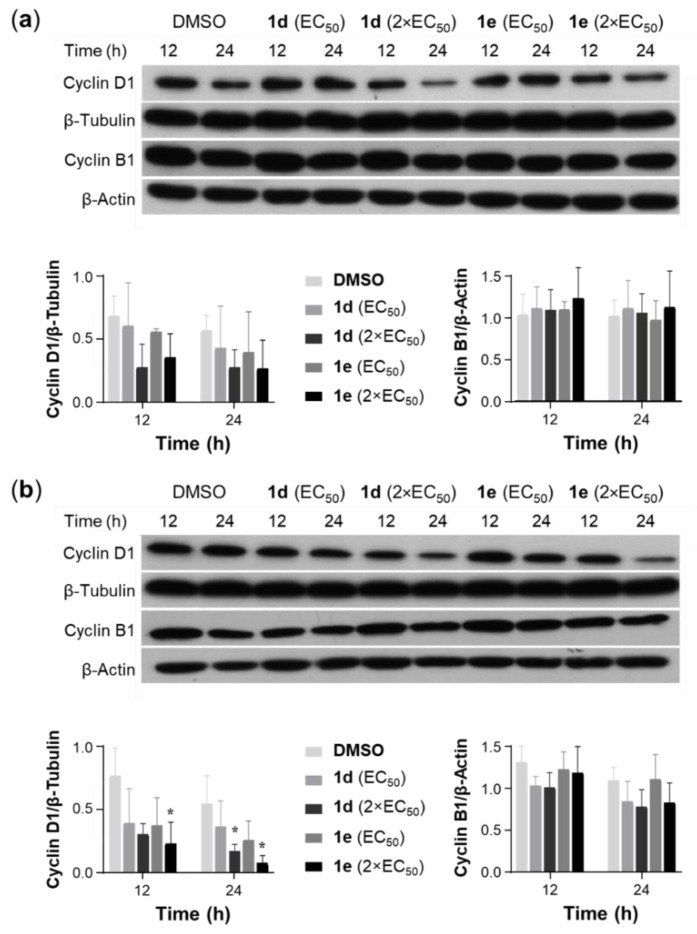
Effect of **1d** and **1e** on the expression of cyclin D1 and B1 in A549 and NCI-H522 cells. Cell lysates of (**a**) A549 and (**b**) NCI-H522 cells, which were treated with **1d** and **1e** at 1× and 2× the EC_50_ concentrations for 12 and 24 h, were subjected to Western blotting with specific antibodies for cyclin D1 and B1. Vehicle control cells were incubated with DMSO (0.5%). Blots shown are representative of *n* = 3. Scanning densitometry of the ratio of cyclin D1 to β-tubulin and cyclin B1 to β-actin in A549 (**a**) and NCI-H522 (**b**) cells. Bars represent mean ± SEM (*n* = 3). Data were analyzed with a two-way ANOVA coupled with a Bonferroni post hoc test. * Indicates significant differences relative to control (*p* < 0.01).

Cell cycle arrest results in cells being no longer involved in the processes of duplication and division [62]. Anticancer agents may act at multiple steps during the cell cycle progression; however, G_1_/S or G_2_/M arrest occur most frequently [63]. We investigated the effects of **1d** and **1e** on cell cycle progression of A549 and NCI-H522 cells using PI staining followed by flow cytometry. At 1× and 2× the EC_50_ concentrations, **1d** and **1e** did not cause any significant changes in the cell cycle distribution of A549 and NCI-H522 cells after 6- and 12-h treatment (Figure S3). Interestingly, **1d** and **1e** produced a weak effect in arresting A549 and NCI-H522 cells at G2/M and G1 phases, respectively, at 12 h. To explore the cell line and time-dependent effects of **1d** and **1e** on cell cycle proteins, the expression of cyclins D1 (a G_1_ progression regulator) and B1 (G_2_/M progression regulator) was investigated. Neither compound induced significant changes in the expression of cyclin D1 and B1 in A549 cells after 12 and 24 h. However, treatment of NCI-H522 cells with **1d** and **1e** at 2× the EC_50_ concentration significantly reduced the expression of cyclin D1 by 68.1% and 84.9% of control after 24 h, while no significant changes in cyclin B1 expression was observed in both NSCLC cells under the same conditions (Figure 7, Figures S4 and S5).

**Figure 8:**
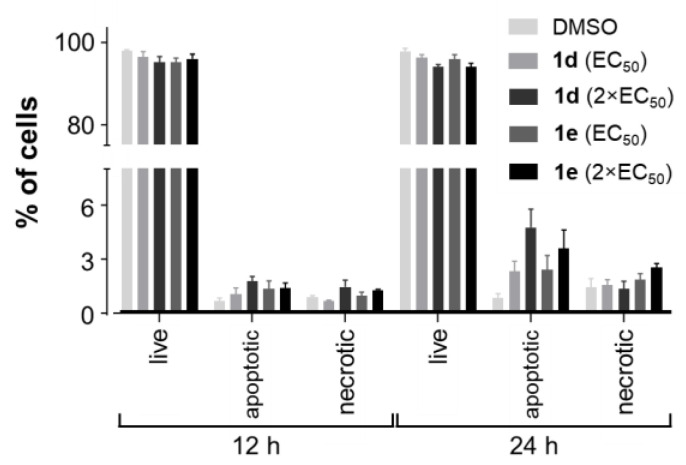
Proportion of apoptotic and necrotic NCI-H522 cells following treatment with **1d** and **1e**. NCI-H522 (3.0 × 10^5^ cells per well) cells were seeded in 6-well plates. NCI-H522 cells were treated with 1× and 2× the EC_50_ of **1d** and **1e** for 12 and 24 h. Vehicle control cells were treated with DMSO (0.5%). Bars indicate the mean number of cells (% of total) ± SEM (*n* = 3). Data were analyzed with a two-way ANOVA coupled with a Bonferroni post hoc test. None were significantly different.

As the previous experiments showed that **1d** and **1e** were more cytotoxic toward NCI-H522 cells compared to A549 cells, the former cell line was chosen to investigate the ability of the compounds to induce apoptosis and/or necrosis after treatment with the anticancer agents for 12 and 24 h. Similar to the results on the cell cycle distribution, no significant changes in the percentage of apoptotic or necrotic NCI-H522 cells were observed following treatment with **1d** and **1e** (Figure 8 and Figure S6). However, it is possible that a higher concentration of the compounds may result in a significant proportion of NCI-H522 cells becoming apoptotic.

## 4. Conclusions

HPs and thio-HPs have interesting biological properties due to their chelating ability to metal centers. In this study, we investigated HP and thio-HP derivatives and their respective Ru(cym)Cl complexes. Both the HP and thio-HP derivatives showed significant antiproliferative activity in a range of cell lines and in all cases the thio-HP compounds were more cytotoxic, which may be due to different affinities for the target. This trend was also observed for the respective Ru(cym)Cl complexes, which, however, were less potent than the organic molecules. Therefore, the most potent derivatives **1d** and **1e** were further investigated and showed higher activity in lung cancer cells compared to breast cancer cells as well as human prostate epithelial cells. While they exhibited cytostatic activity in A549 cells, they were cytotoxic in NCI-H522 cells. Experiments on the aggressiveness of A549 and NCI-H522 cell growth revealed no effect of withdrawal of the compounds on the cell number. In clonogenic assays, it was shown that the compounds did not prevent colony formation, nor were **1d** and **1e** effective as HDAC inhibitors excluding HDACs as potential targets. Lastly, they did not significantly modulate cell cycle progression of NSCLCs, while concentration-dependent apoptosis induction ability was confirmed.

## Figures and Tables

**Table 1:** 50% inhibitory concentration (IC_50_) values of (thio)pyridones **1a**–**1f** and Ru complexes **2a**–**2f** following incubation for 72 h on human colorectal cancer (HCT116), non-small cell lung (NCI-H460), cervical (SiHa), and colon cancer (SW480) cell lines.

Compound	IC_50_/µM			
	HCT116	NCI-H460	SW480	SiHa
**1a**	11 ± 2	10 ± 1	13 ± 0.1	25 ± 1
**1b**	6.3 ± 5.3	7.0 ± 2.1	9.8 ± 1.1	24 ± 8
**1c**	5.4 ± 0.02	4.2 ± 0.3	5.3 ± 1.0	14 ± 0.1
**1d**	0.16 ± 0.04	0.42 ± 0.07	0.28 ± 0.04	0.74 ± 0.06
**1e**	0.06 ± 0.01	0.18 ± 0.05	0.04 ± 0.03	0.40 ± 0.02
**1f**	0.17 ± 0.08	1.5 ± 2.5	3.6 ± 6.2	0.50 ± 0.02
**2a**	16 ± 2	13 ± 1	14 ± 0.3	25 ± 1
**2b**	11 ± 1	9.5 ± 3.4	11 ± 4	23 ± 6
**2c**	13 ± 0.4	13 ± 1	16 ± 0.4	34 ± 0.1
**2d**	0.35 ± 0.04	0.94 ± 0.06	0.50 ± 0.22	1.6 ± 0.1
**2e**	0.48 ± 0.02	1.3 ± 0.1	1.1 ± 0.1	1.4 ± 0.1
**2f**	0.35 ± 0.03	0.90 ± 0.13	0.79 ± 0.13	1.5 ± 0.0
SAHA	0.46 ± 0.09	0.57 ± 0.01	1.3 ± 0.1	1.6 ± 0.1

**Table 2:** Cytotoxic activity (mean half-maximal effective concentration (EC_50_) ± SEM) of **1d** and **1e** and their Ru complexes **2d** and **2e** as compared to SAHA in human lung (A549, NCI-H522), breast (MDA-MB-231, MDA-MB-468), and prostate (PC3) cancer cell lines. EC_50_ values were determined using the SRB assay after 72 h of treatment.

Compound	EC_50_/µM				
	A549	NCI-H522	MDA-MB-231	MDA-MB-468	PC3
**1d**	0.36 ± 0.01	0.28 ± 0.01	2.79 ± 0.14	1.70 ± 0.19	0.33 ± 0.04
**1e**	0.32 ± 0.01	0.23 ± 0.02	2.64 ± 0.11	3.33 ± 0.63	1.44 ± 0.21
**2d**	1.73 ± 0.02	6.67 ± 0.30	7.24 ± 0.31	5.46 ± 0.41	7.93 ± 0.25
**2e**	1.66 ± 0.06	7.42 ± 0.19	7.28 ± 0.51	8.70 ± 0.51	11.11 ± 0.89
SAHA	1.01 ± 0.05	0.35 ± 0.02	2.01 ± 0.11	5.56 ± 0.64	1.13 ± 0.19

**Table 3:** Mean values for residual activity of HDAC8 after treatment with **1a, 1b, 1d, 1e, 2a, 2b, 2d**, and **2e** at 10 μM. The numbers in brackets are the two recorded data points (*n* = 2).

Compound	HDAC8 Activity/%
**1a**	107 (110,104)
**1b**	108 (107,108)
**2a**	59 (58,59)
**2b**	94 (96,92)
**1d**	52 (53,50)
**1e**	30 (32,28)
**2d**	23 (23,23)
**2e**	35 (35,34)
SAHA, IC_50_ = 0.84 ± 0.02 μM [61]	

## Data Availability

The data presented in this study are available in this article and supplementary material.

## Supplementary Materials

The following are available online at https://www.mdpi.com/2227-9059/9/2/123/s1. Tables S1 and S2. X-ray diffraction analysis measurement parameters and bond lengths [Å] and angles [°] for 1b and 1d. Table S3. Selectivity index (SI) of potent hydroxythiopyridone derivatives (1d and 1e) in different human cancer cell lines. Figures S1 and S2. X-ray diffraction results and molecular structures. Figures S3–S6. Cell biological studies and additional data. Figures S7–S21. NMR spectra.
