# Supplementary material for: High Antiproliferative Activity of Hydroxythiopyridones over Hydroxypyridones and Their Organoruthenium Complexes
Source: Biomedicines. 2021 Jan 27;9(2):123. doi: 10.3390/biomedicines9020123 (PMC7912191; doi:10.3390/biomedicines9020123)
Supplement: Supplementary file 1 [file biomedicines-09-00123-s001.pdf]

biomedicines

Supporting Information to

High Antiproliferative Activity of Hydroxythiopyridones Over Hydroxypyridones and their Organoruthenium Complexes

Md. Salman Shakil [1,#], Shahida Parveen [2,3,#], Zohaib Rana [1], Fearghal Walsh [2], Sanam Movassaghi [2], Tilo Söhnel [2], Mayur Azam [1], Muhammad Ashraf Shaheen [3], Stephen M.F. Jamieson [4], Muhammad Hanif [2,*], Rhonda J. Rosengren [1,*], and Christian G. Hartinger [2,*]

[1] Department of Pharmacology and Toxicology, University of Otago, Dunedin 9016, Dunedin, New Zealand.
[2] School of Chemical Sciences, University of Auckland, Private Bag 92019, Auckland 1142, New Zealand, http://hartinger.auckland.ac.nz/
[3] Department of Chemistry, University of Sargodha, Sargodha 40100, Pakistan.
[4] Auckland Cancer Society Research Centre, University of Auckland, Private Bag 92019, Auckland 1142, New Zealand.
[#] These authors contributed equally to this work.
* Correspondence: m.hanif@auckland.ac.nz (M.H.); rhonda.rosengren@otago.ac.nz (R.J.R.); c.hartinger@auckland.ac.nz (C.G.H.)

Table of Contents

Additional XRD, NMR spectroscopic and cell biological data

Table S1. X-ray diffraction analysis measurement parameters.

	**1b**·MeOH	**1d**
CCDC	2049245	2049246
Empirical formula	$C_{15}H_{19}NO_3$	$C_{13}H_{13}NOS$
Formula weight / g mol^{-1}	261.31	231.32
Temperature / K	100	100
Crystal system	triclinic	triclinic
Space group	*P*-1	*P*-1
a / Å	7.5960(3)	6.9685(3)
b / Å	10.0395(3)	8.2820(3)
c / Å	10.4920(4)	11.1698(4)
α / °	112.378(2)	110.175(2)
β / °	107.967(2)	93.567(2)
γ / °	94.975(2)	108.548(2)
Volume / Å^3	684.56(4)	562.85(4)
Z	2	2
ρ_{calc} / g cm^{-3}	1.268	1.365
μ / mm^{-1}	0.088	0.264
F(000)	280.0	244.0
Crystal size / mm^3	0.28 × 0.14 × 0.12	0.4 × 0.28 × 0.15
Radiation	MoKα (λ = 0.71073)	MoKα (λ = 0.71073)
2Θ range for data collection / °	5.796 to 50.498	5.49 to 50.5
Index ranges	-9 ≤ h ≤ 9	-8 ≤ h ≤ 8
	-12 ≤ k ≤ 12	-9 ≤ k ≤ 9
	-12 ≤ l ≤ 12	-13 ≤ l ≤ 13
Reflections collected	12123	10428
Independent reflections	2472 [R_{int} = 0.0537,	2028 [R_{int} = 0.0428,
	R_{sigma} = 0.0411]	R_{sigma} = 0.0300]
Data/restraints/parameters	2472/0/179	2028/0/147
Goodness-of-fit on F^2	1.030	1.088
Final R indexes [I>=2σ (I)]	R_1 = 0.0384, wR_2 = 0.0900	R_1 = 0.0320, wR_2 = 0.0877
Final R indexes [all data]	R_1 = 0.0559, wR_2 = 0.0996	R_1 = 0.0337, wR_2 = 0.0896
Largest diff. peak/hole / e Å^{-3}	0.26/-0.18	0.28/-0.25

Table S2. Selected bond lengths [Å] and angles [°] for **1b** and **1d**.

**Bond lengths Å / angles °**	**1b**	**1d**
C4–O2/S	1.2802(18)	1.7188(15)
C3–O1	1.3640(17)	1.3582(17)
C3–C4	1.428(2)	1.421(2)
C2–C3	1.373(2)	1.378(2)
O2/S–C4–C3–O2	1.93	0.57

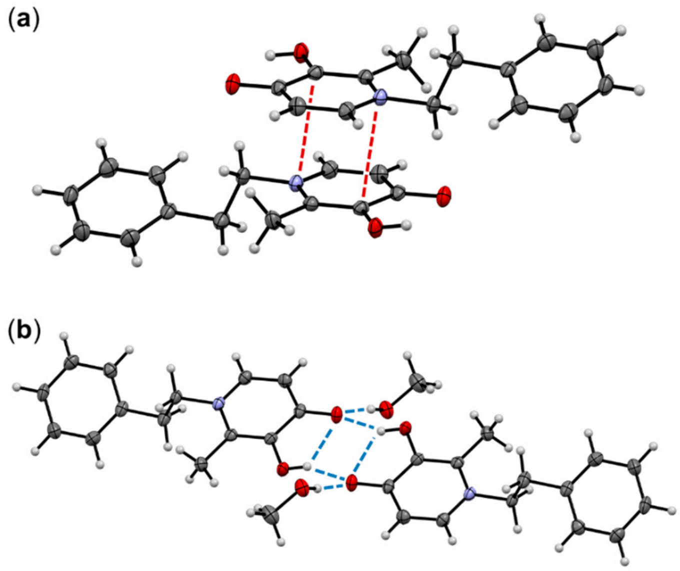

Figure S1. (**a**) π-stacking interaction found in the molecular structure of **1b** with the shortest distance at 3.312 Å indicated as dashed, red lines; (**b**) Inter- and intramolecular H bond formation between two molecules of **1b** and co-crystallized methanol indicated as a dashed, blue lines.

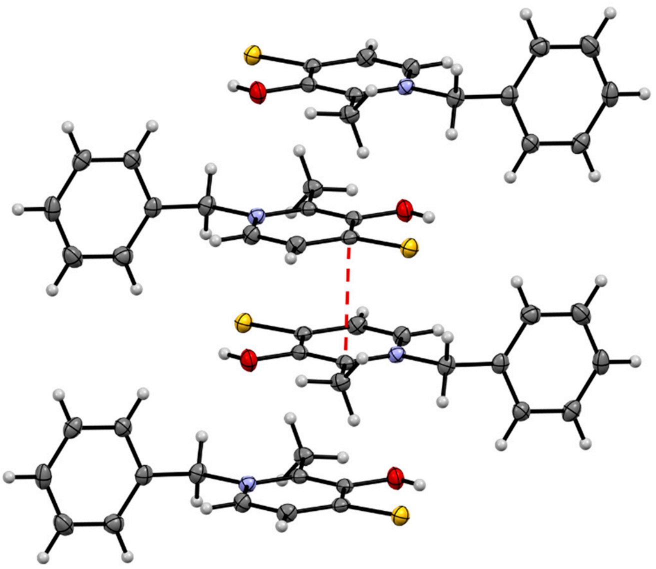

Figure S2. Stacking of four molecules of **1d** and π-stacking interaction found between two molecules of **1d** with the shortest distance at 3.523 Å indicated as a dashed, red line.

Table S3. Selectivity index (SI) of potent hydroxythiopyridone derivatives (**1d** and **1e**) in different human cancer cell lines. SI values were calculated considering human prostate epithelial PNT1A cell line as normal cells.

**Compound**	**EC_{50} (μM) PNT1A**	**Selectivity Index**				
		**A549**	**NCI-H522**	**MDA-MB-231**	**MDA-MB-468**	**PC3**
**1d**	1.29 ± 0.06	3.58	4.61	0.46	0.75	3.91
**1e**	1.12 ± 0.02	3.50	4.86	0.42	0.33	0.77

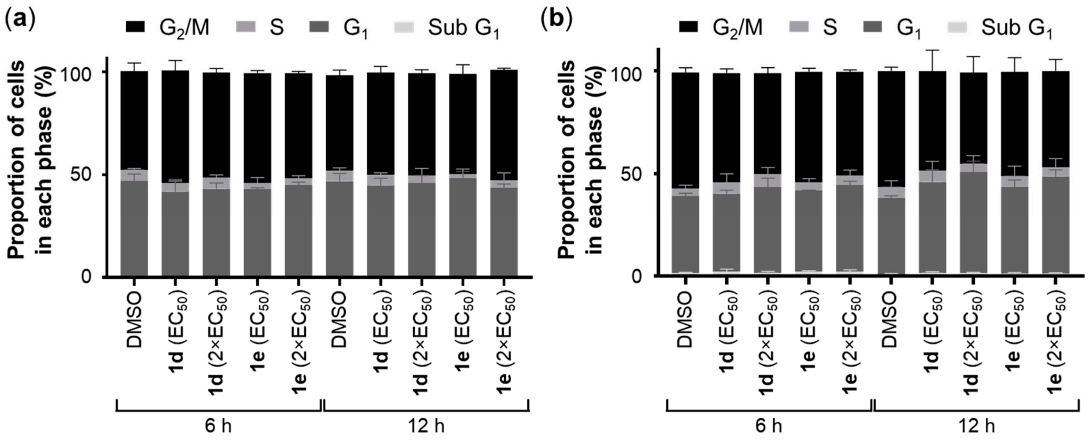

Figure S3. Cell cycle analysis in A549 and NCI-H522 cells exposed to **1d** and **1e.** A549 (1 × 10^6 cells per dish) cells were seeded in 10 cm cell culture dishes and NCI-H522 (3.0 × 10^5 cells per well) cells were seeded in 6-well plates and left to attach for 24 h at 37 °C. (**a**) A549 cells were treated with 0.72 μM of **1d** and 0.64 μM of **1e** while (**b**) NCI-H522 cells were treated with 0.56 μM of **1d** and 0.46 μM of **1e**, both for 6 and 12 h. Vehicle control cells were incubated with DMSO (0.5%). Bars indicate the mean proportion of cells in the different cell cycle phases (% of total) ± SEM (n = 3). Data were analyzed with a two-way ANOVA coupled with a Bonferroni post-hoc test. No statistical significances were observed ($p < 0.01$).

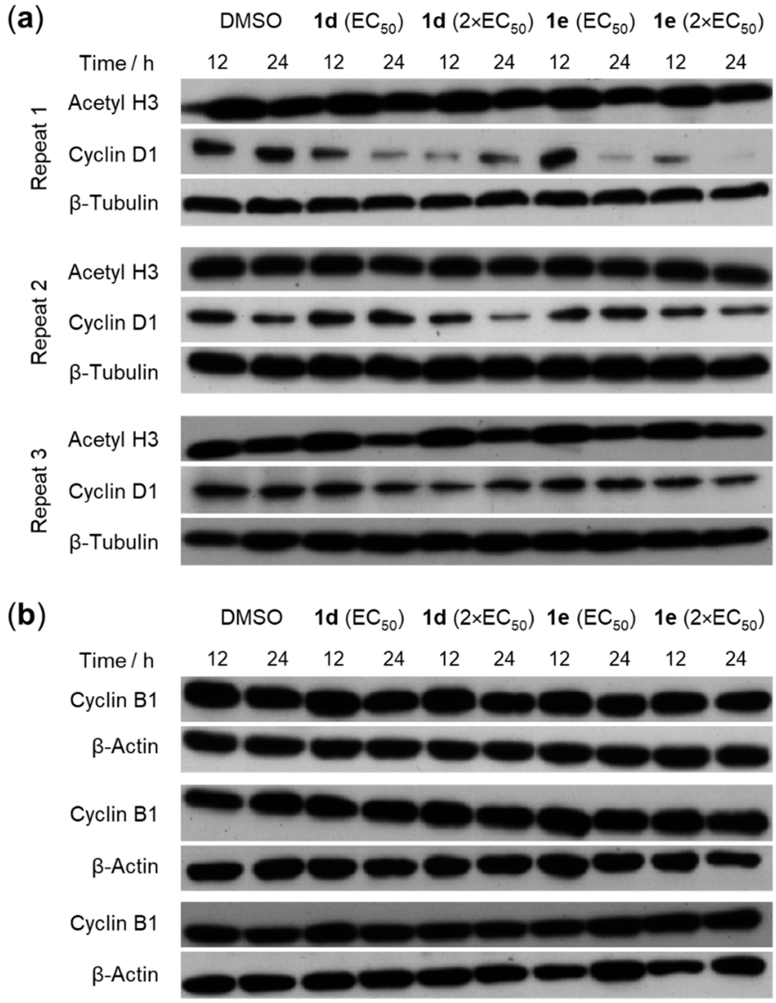

Figure S4. Effect of **1d** and **1e** on (**a**) acetyl-H3, and cyclin D1 and (**b**) B1 expression in A549 cells.

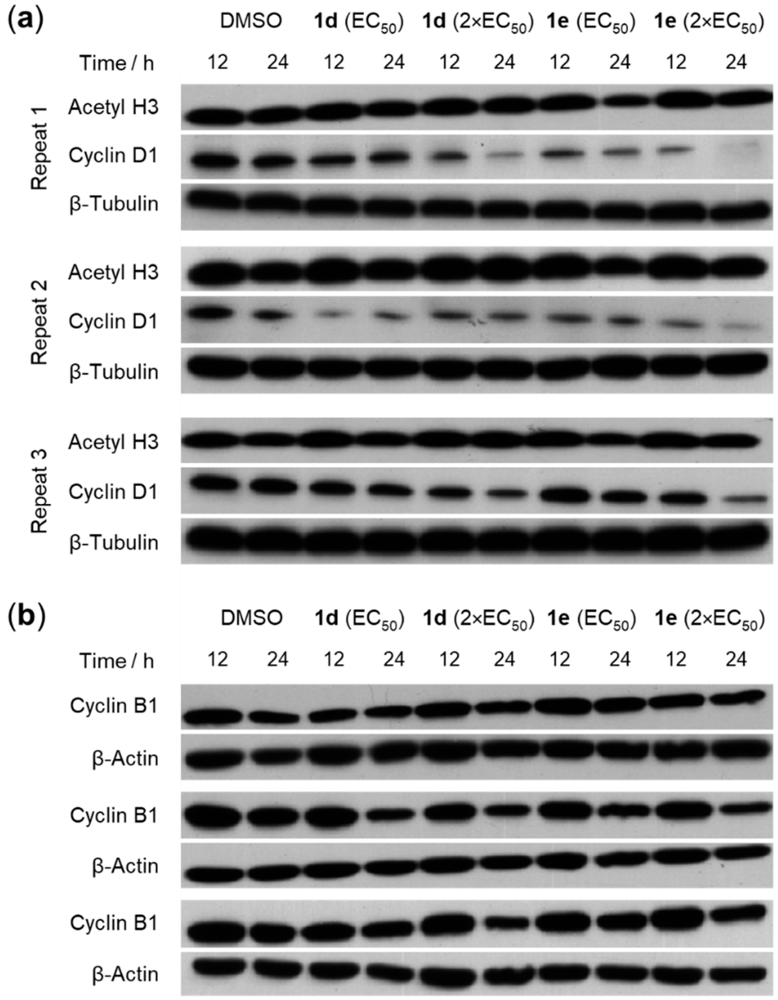

Figure S5. Effect of **1d** and **1e** on (**a**) acetyl-H3, and cyclin D1 and (**b**) B1 expression in NCI-H522 cells.

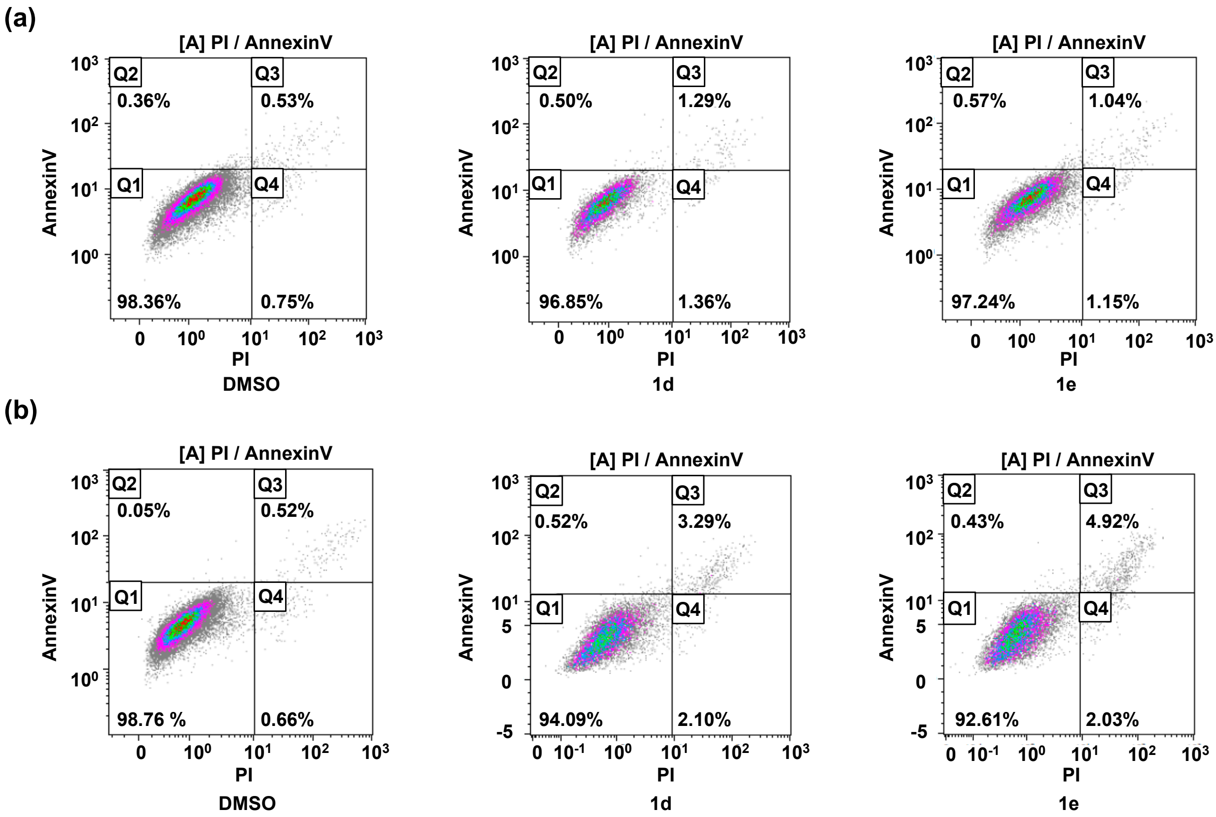

Figure S6. Number of live, apoptotic and necrotic NCI-H522 cells following treatment with **1d** and **1e**. NCI-H522 (3.0×10^5 cells per well) cells were seeded in 6-well plates. Representative flow cytometry image of live (Q1), apoptotic (early apoptotic: Q2; late apoptotic: Q3) and necrotic (Q4) NCI-H522 cells were treated with 2× the EC_{50} of **1d** and **1e** for 12 h (a) and 24 h (b). Vehicle control cells were treated with DMSO (0.5%). PI: Propidium iodide.

NMR spectra

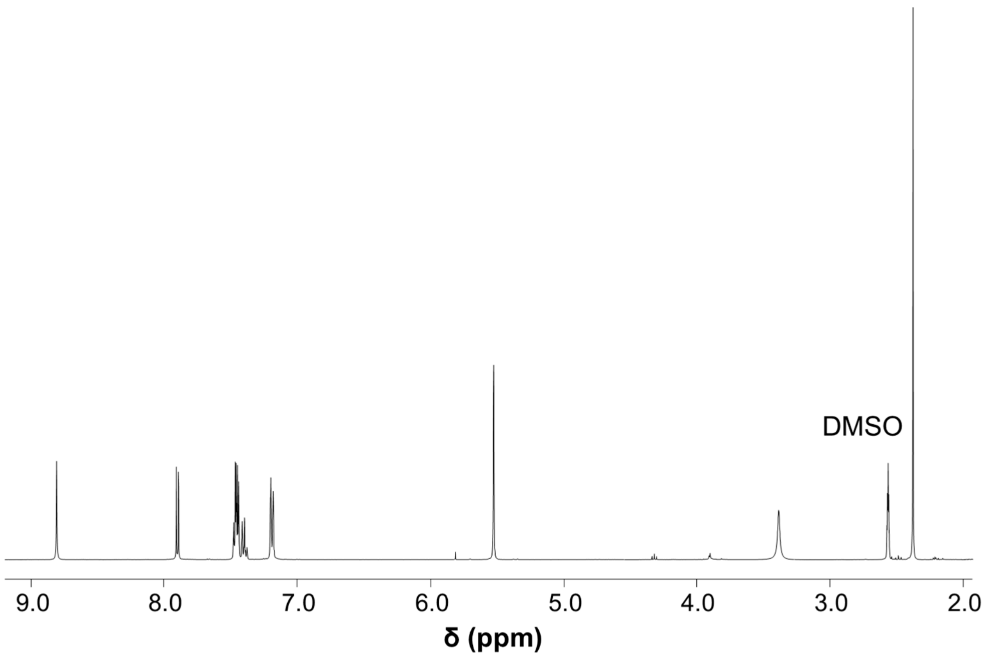

Figure S7. ^{1}H NMR spectrum of **1d** in d_6-DMSO.

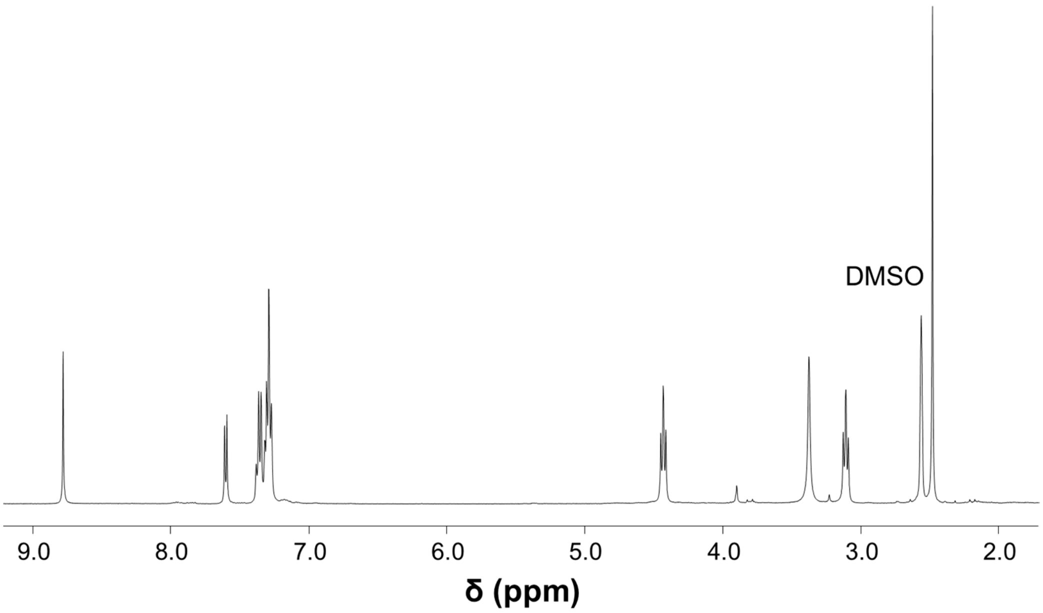

Figure S8. ^{1}H NMR spectrum of **1e** in d_6-DMSO.

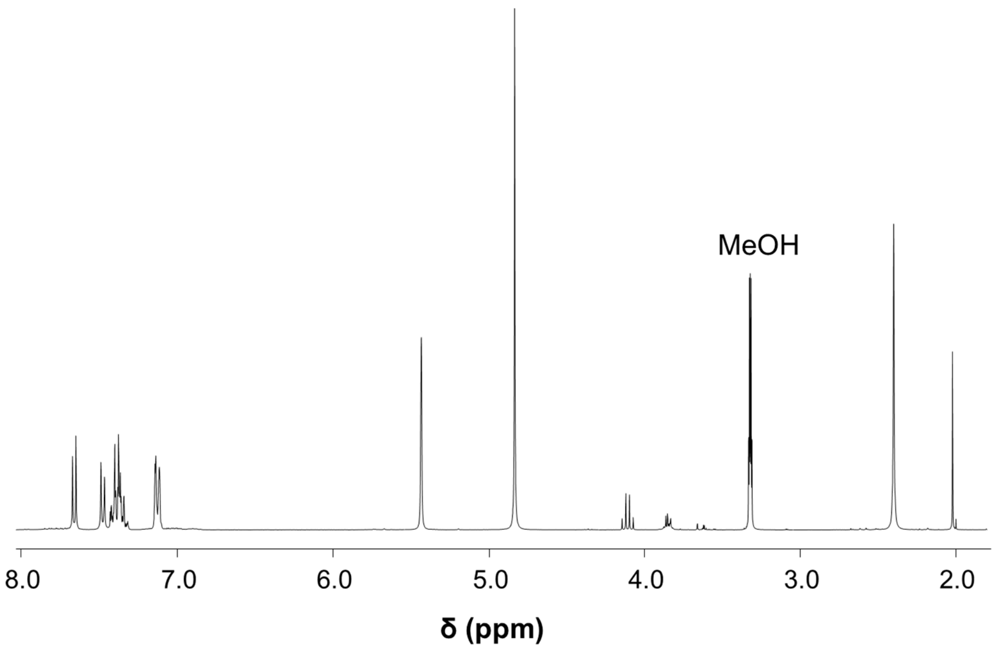

Figure S9. ^{1}H NMR spectrum of **1f** in d_4-MeOD.

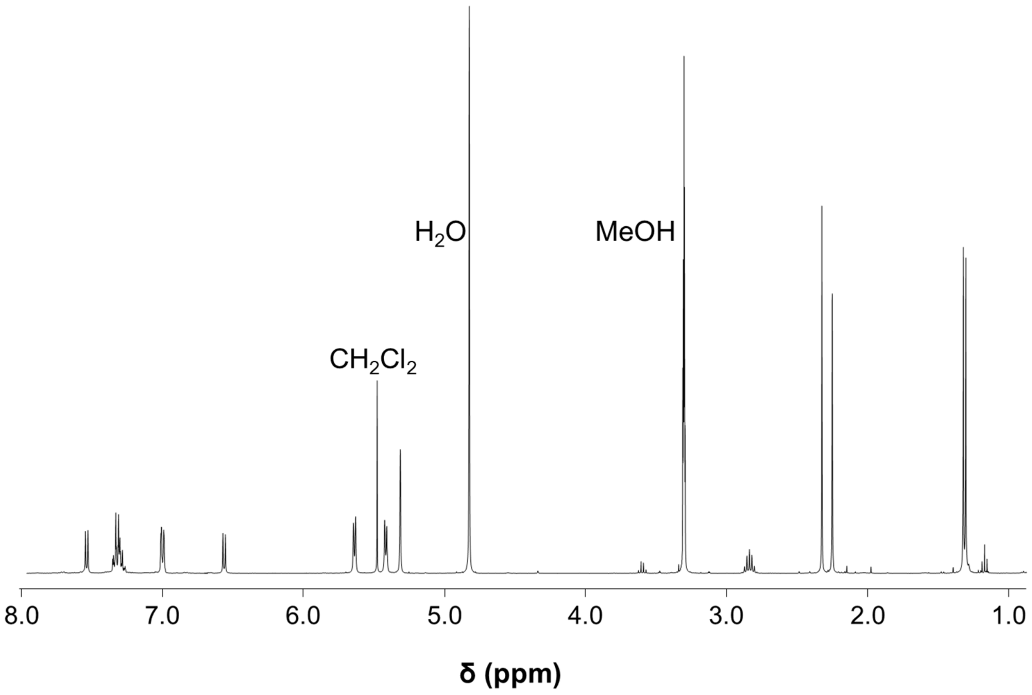

Figure S10. ^{1}H NMR spectrum of **2a** in d_4-MeOD.

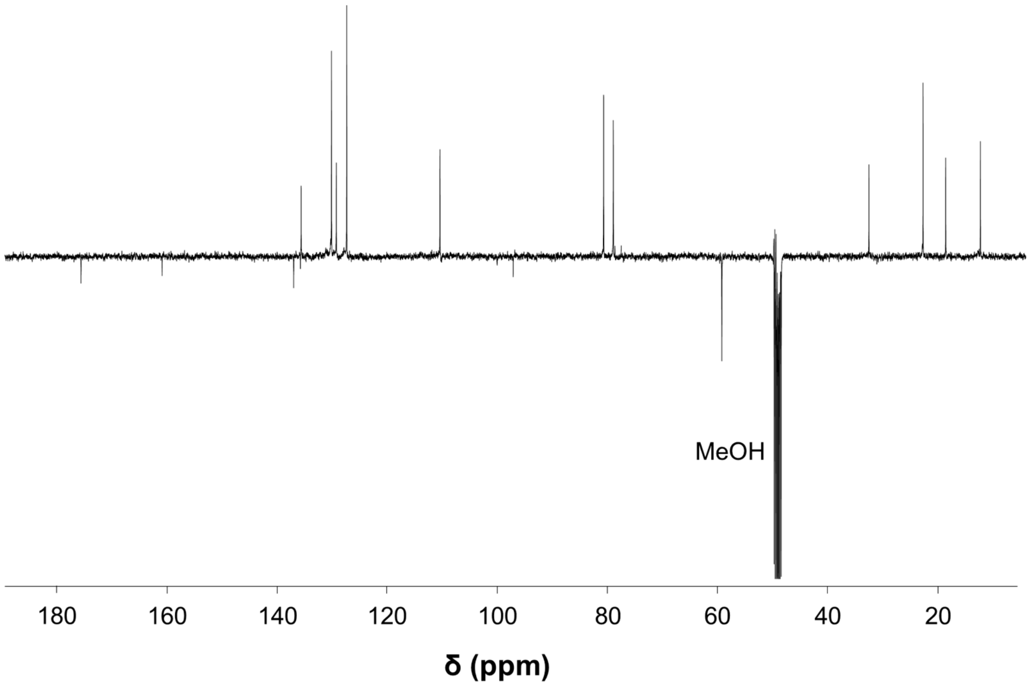

Figure S11. $^{13}C\{^{1}H\}$ NMR spectrum of **2a** in d_4-MeOD.

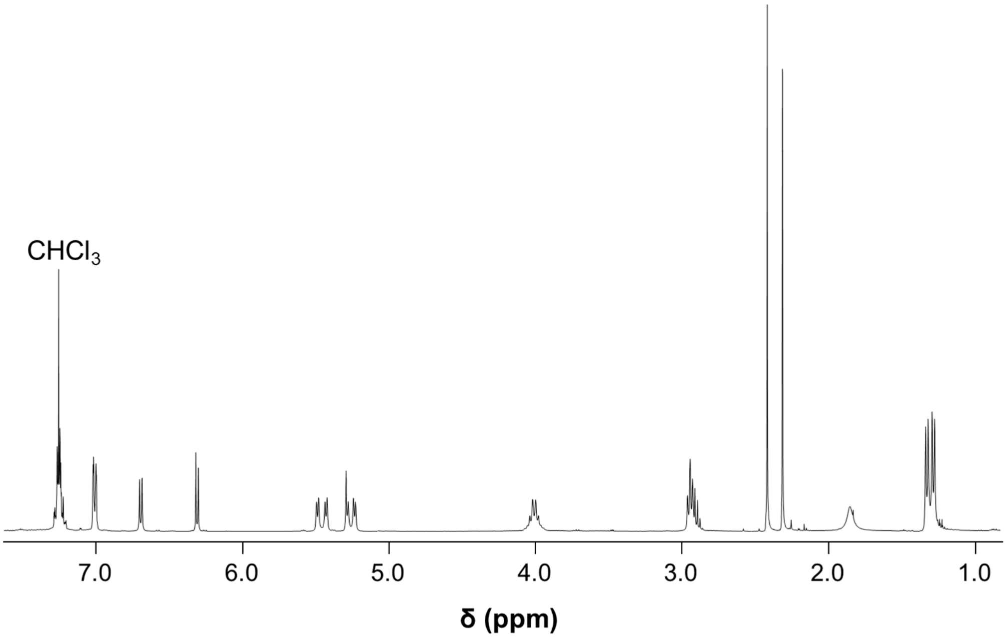

Figure S12. ^{1}H NMR spectrum of **2b** in $CDCl_3$.

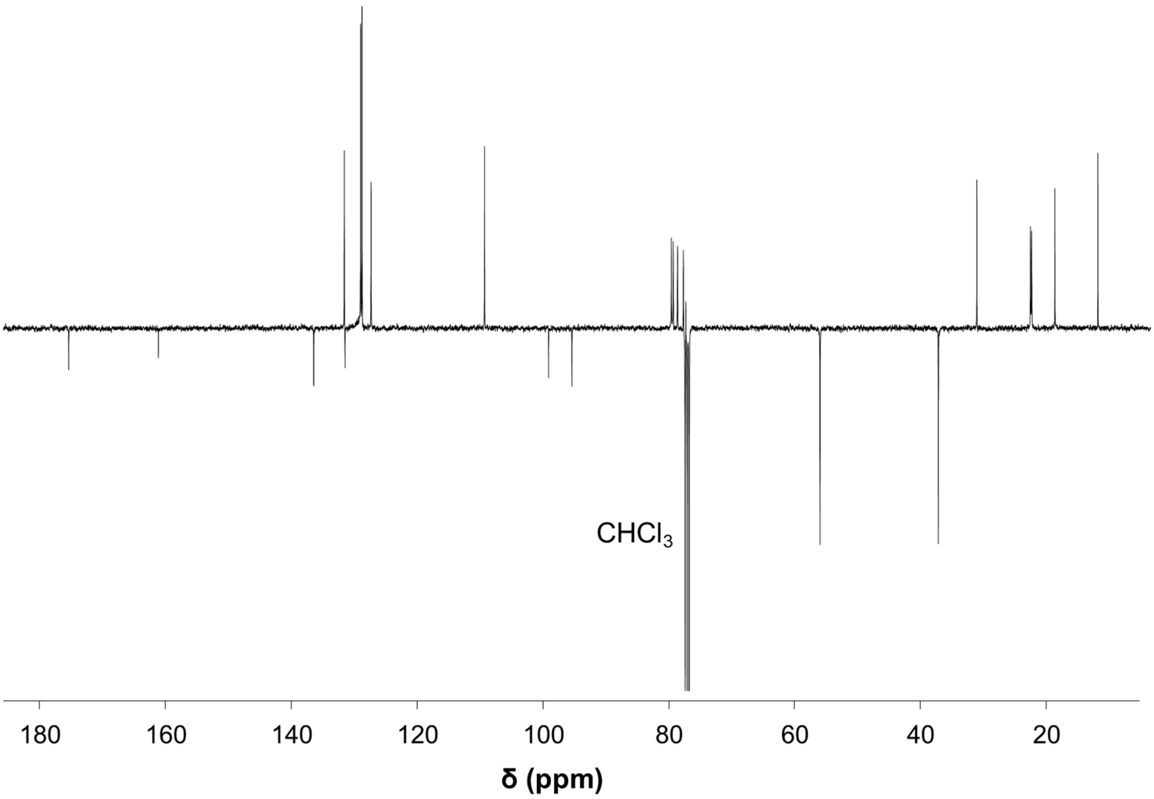

Figure S13. $^{13}C\{^{1}H\}$ NMR spectrum of **2b** in $CDCl_3$.

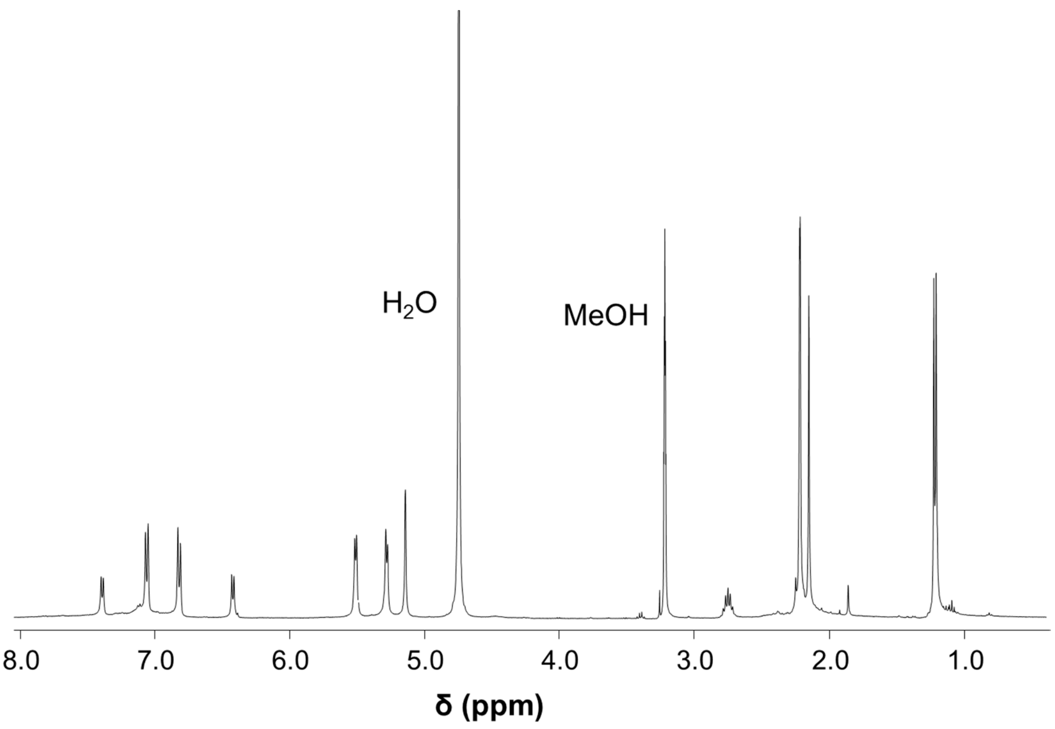

Figure S14. ^{1}H NMR spectrum of **2c** in d_4-MeOD.

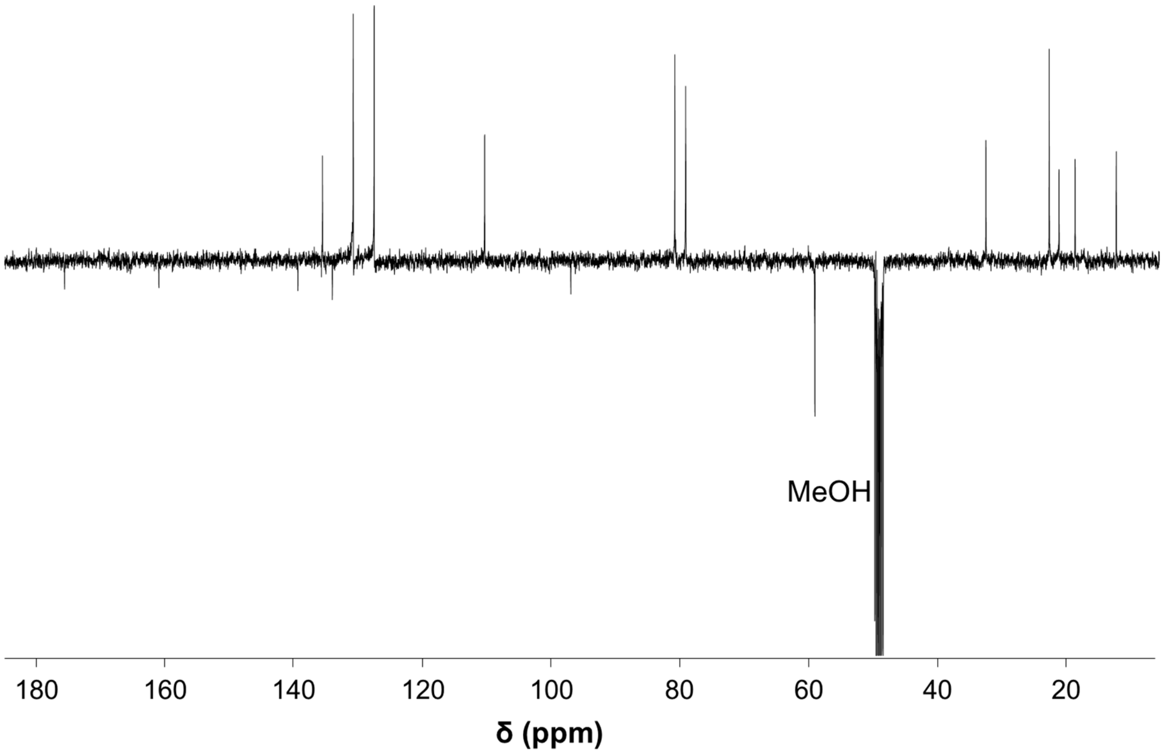

Figure S15. ^{13}C{^{1}H} NMR spectrum of **2c** in d_4-MeOD.

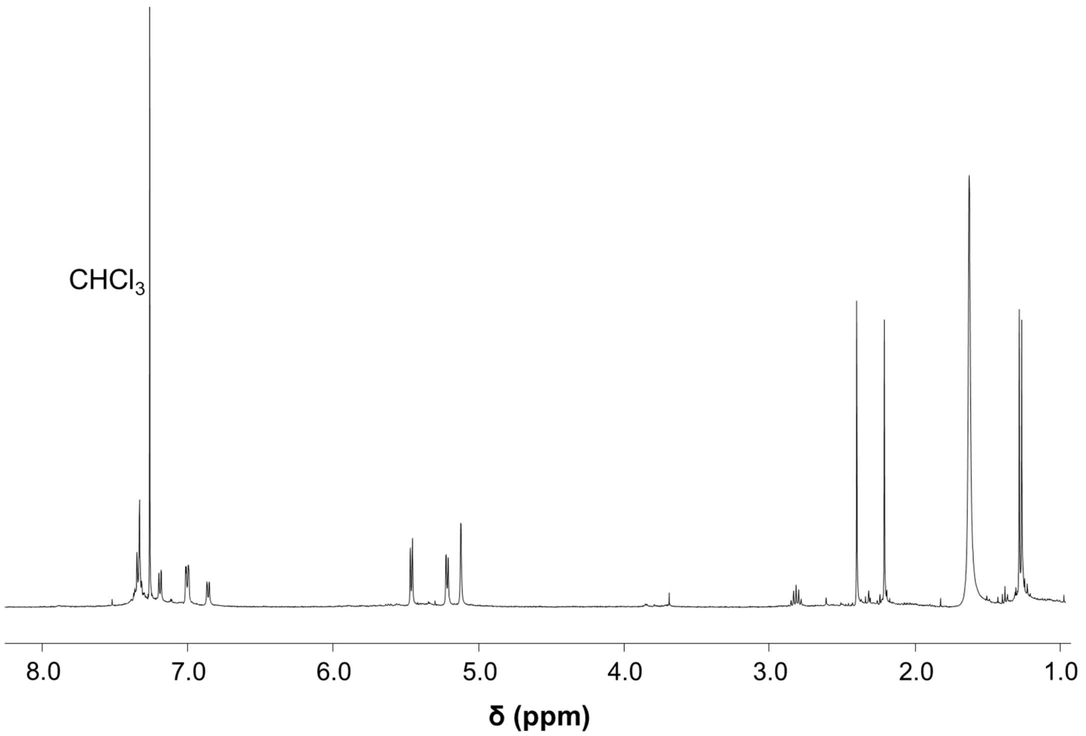

Figure S16. ^{1}H NMR spectrum of **2d** in $CDCl_3$.

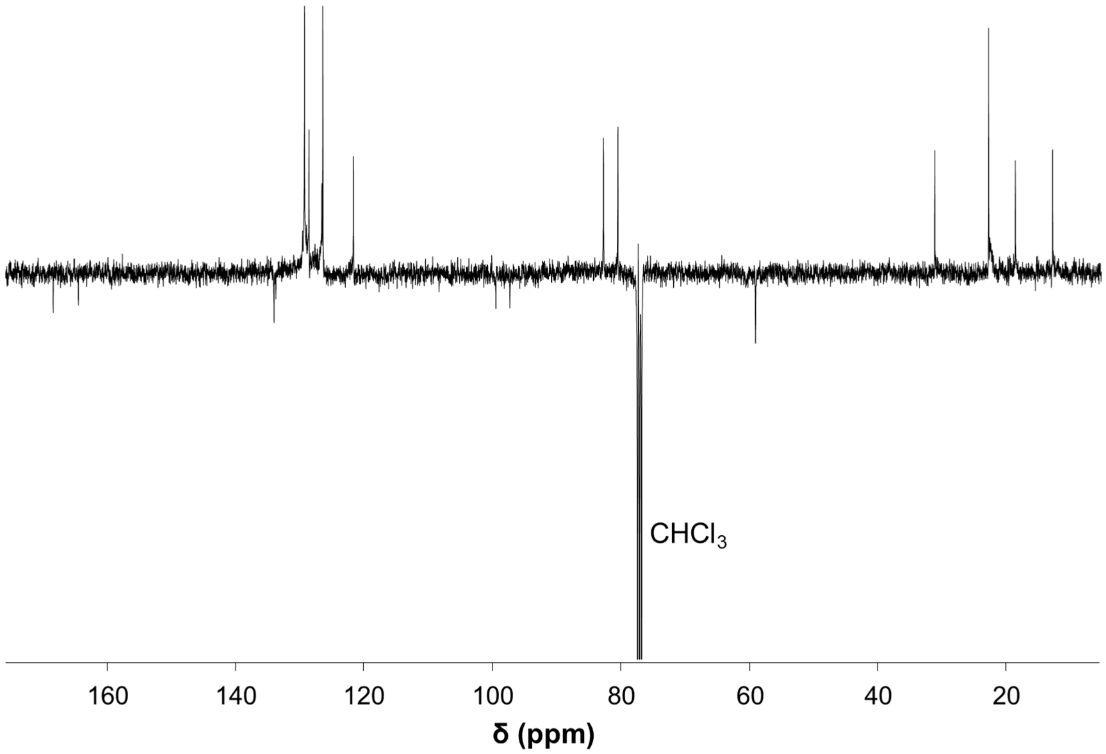

Figure S17. $^{13}C\{^{1}H\}$ NMR spectrum of **2d** in $CDCl_3$.

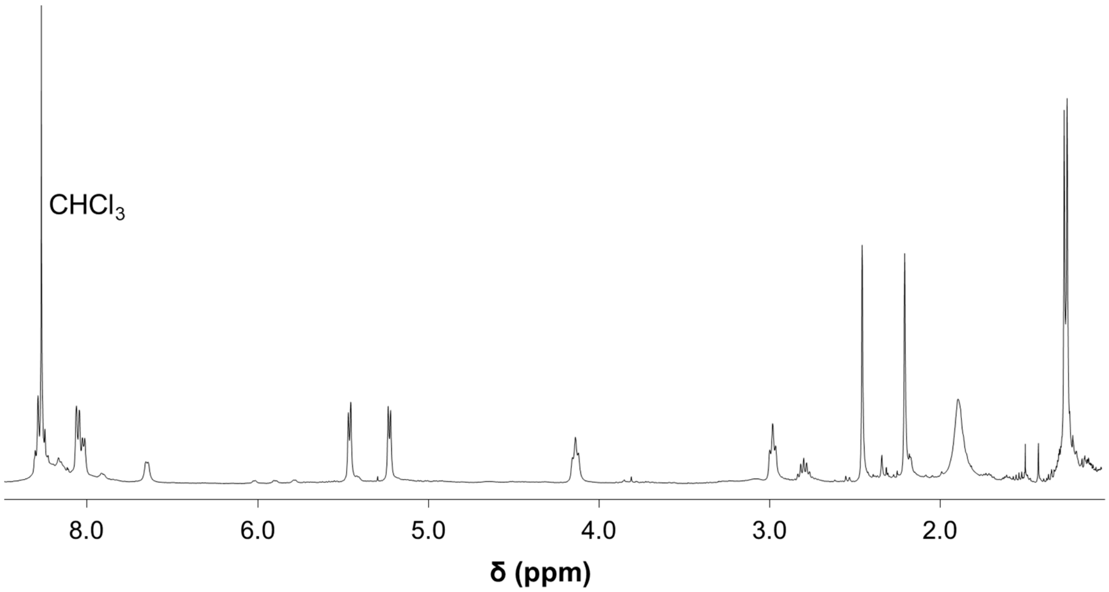

Figure S18. ^{1}H NMR spectrum of **2e** in $CDCl_3$.

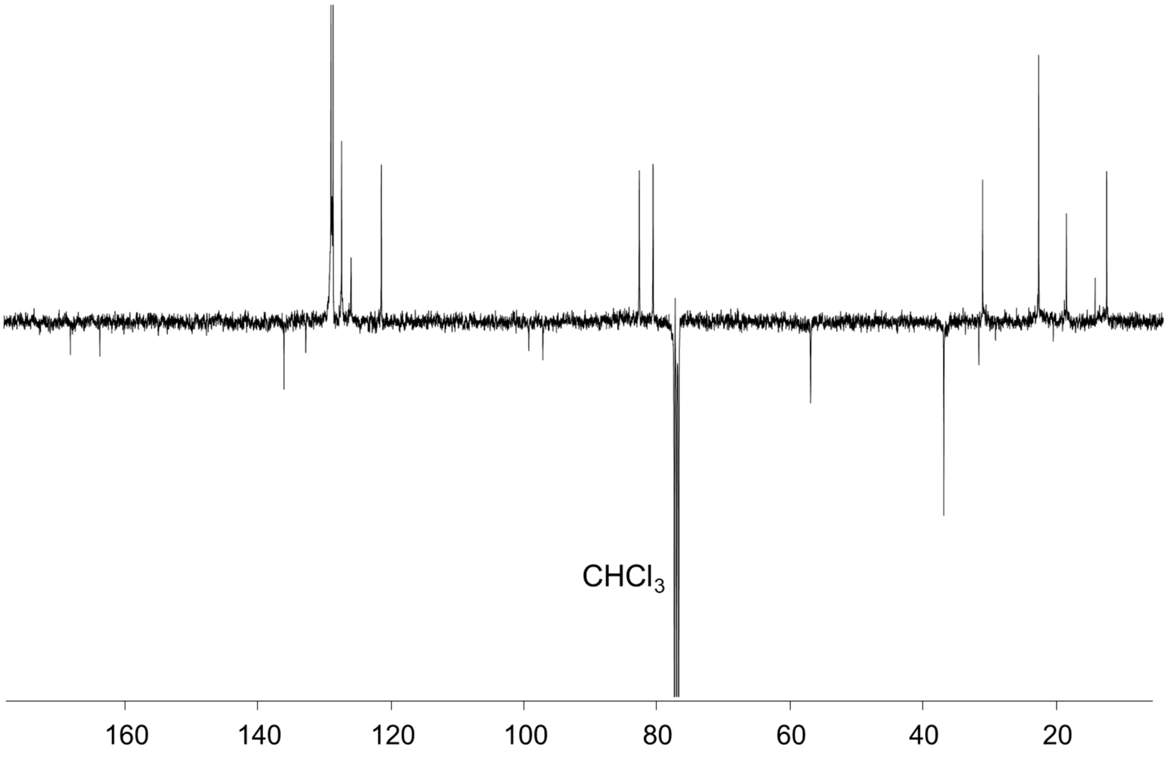

Figure S19. $^{13}C\{^{1}H\}$ NMR spectrum of **2e** in $CDCl_3$.

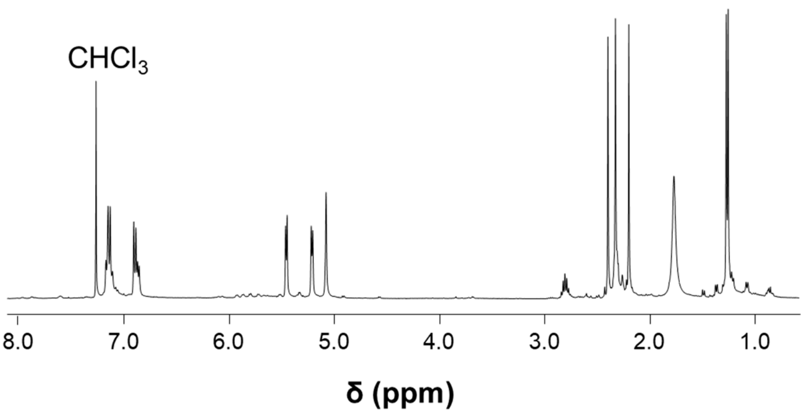

Figure S20. ^{1}H NMR spectrum of **2f** in $CDCl_3$.

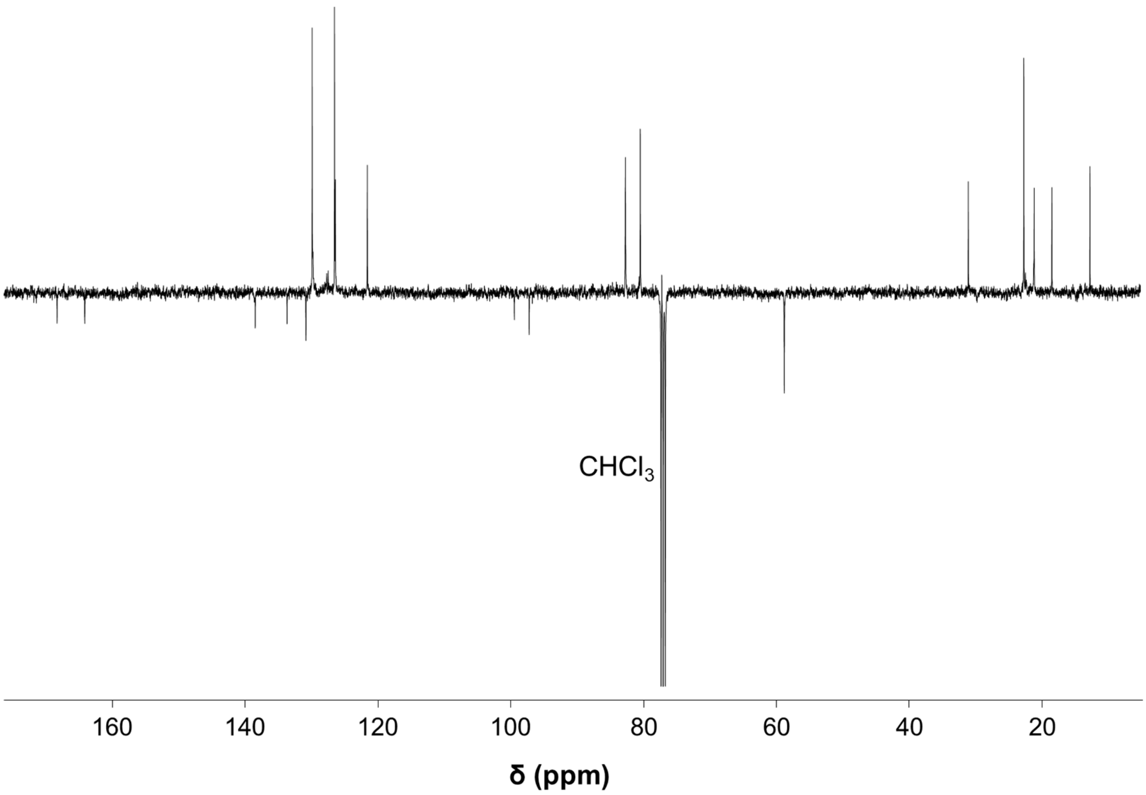

Figure S21. $^{13}C\{^{1}H\}$ NMR spectrum of **2f** in $CDCl_3$.

© 2020 by the authors. Submitted for possible open access publication under the terms and conditions of the Creative Commons Attribution (CC BY) license (http://creativecommons.org/licenses/by/4.0/).
